# Galectin-3 Released by Pancreatic Ductal Adenocarcinoma Suppresses γδ T Cell Proliferation but Not Their Cytotoxicity

**DOI:** 10.3389/fimmu.2020.01328

**Published:** 2020-06-30

**Authors:** Daniel Gonnermann, Hans-Heinrich Oberg, Marcus Lettau, Matthias Peipp, Dirk Bauerschlag, Susanne Sebens, Dieter Kabelitz, Daniela Wesch

**Affiliations:** ^1^Institute of Immunology, University Hospital Schleswig-Holstein (UKSH) and Christian-Albrechts University (CAU) of Kiel, Kiel, Germany; ^2^Division of Stem Cell Transplantation and Immunotherapy, Department of Medicine II, UKSH, CAU Kiel, Kiel, Germany; ^3^Department of Gynecology and Obstetrics, UKSH, Kiel, Kiel, Germany; ^4^Institute for Experimental Cancer Research, UKSH, CAU Kiel, Kiel, Germany

**Keywords:** T cells, gammadelta T cells, pancreatic cancer, galectin-3, α3β1 integrin, bispecific antibodies, proliferation, autologous

## Abstract

Pancreatic ductal adenocarcinoma (PDAC) is characterized by an immunosuppressive tumor microenvironment with a dense desmoplastic stroma. The expression of β-galactoside-binding protein galectin-3 is regarded as an intrinsic tumor escape mechanism for inhibition of tumor-infiltrating T cell function. In this study, we demonstrated that galectin-3 is expressed by PDAC and by γδ or αβ T cells but is only released in small amounts by either cell population. Interestingly, large amounts of galectin-3 were released during the co-culture of allogeneic *in vitro* expanded or allogeneic or autologous resting T cells with PDAC cells. By focusing on the co-culture of tumor cells and γδ T cells, we observed that knockdown of galectin-3 in tumor cells identified these cells as the source of secreted galectin-3. Galectin-3 released by tumor cells or addition of physiological concentrations of recombinant galectin-3 did neither further inhibit the impaired γδ T cell cytotoxicity against PDAC cells nor did it induce cell death of *in vitro* expanded γδ T cells. Initial proliferation of resting peripheral blood and tumor-infiltrating Vδ2-expressing γδ T cells was impaired by galectin-3 in a cell-cell-contact dependent manner. The interaction of galectin-3 with α3β1 integrin expressed by Vδ2 γδ T cells was involved in the inhibition of γδ T cell proliferation. The addition of bispecific antibodies targeting γδ T cells to PDAC cells enhanced their cytotoxic activity independent of the galectin-3 release. These results are of high relevance in the context of an *in vivo* application of bispecific antibodies which can enhance cytotoxic activity of γδ T cells against tumor cells but probably not their proliferation when galectin-3 is present. In contrast, adoptive transfer of *in vitro* expanded γδ T cells together with bispecific antibodies will enhance γδ T cell cytotoxicity and overcomes the immunosuppressive function of galectin-3.

## Introduction

Galectin (gal)-3 is a member of β-galactoside-binding protein family that shares highly conserved carbohydrate recognition domains (CRD) (1, 2). The monomer gal-3 belongs to the chimera-type subgroup of the galectin family which contains one CRD that is connected to an extended non-lectin N-terminal domain. This exclusive gal-3 structure allows dimerization in the absence of binding ligands and a formation of pentamers in the presence of carbohydrate binding ligands such as N-glycans (3). As a multifunctional protein, gal-3 is involved in cell-matrix adhesion, cell proliferation, cell death, receptor turnover, and cell signaling as well as in malignant transformation depending on its subcellular localization (1–4). Gal-3 is found in the cytoplasm, shuttles between the cytoplasm and the nucleus, and can also be expressed at the cell surface or secreted into biological fluids *via* non-classical secretory pathways (3). Depending on the cellular component, gal-3 mediates both pro-and anti-apoptotic activity (5). Gal-3 overexpression as well as prominent protumorigenic effects have been shown in various tumors including pancreatic ductal adenocarcinoma (PDAC) (6). Differential expression profiling and microarray analysis revealed an enhanced gal-3 expression in the tissue of PDAC patients compared to that of chronic pancreatitis (CP) patients, and a slightly increased gal-3 expression in tissue of CP patients compared to healthy donors (7–9).

PDAC is 4th leading cancer-related death due to an aggressive growth, early metastatic dissemination and limited treatment options (10, 11). Mutations in the pro-oncogene K-Ras (rat sarcoma) together with a high Ras activity are suggested to be associated with the pathogenesis of PDAC (12, 13). An overexpression of gal-3 in pancreatic tumor tissue contributes to PDAC progression *via* gal-3 binding to retaining Ras at the plasma membrane maintaining Ras-signaling including phosphorylation of Extracellular-signal Regulated Kinases (ERK) and AKT and Ras-like (Ral) protein A activity (12–14).

In addition to the gal-3-mediated tumor transformation, gal-3 secreted by tumor cells regulates immune cell activities and contributes to immunosuppression (15). Extracellular gal-3 binds glycosylated T cell surface receptors including the receptor-linked protein tyrosine phosphatase CD45 expressed on all leukocytes, integrins like CD11a (αL integrin), CD29 (β1 integrin), and CD49c (α3 integrin) and the T cell interaction molecule CD7 (1, 16). Cross-linking glycoproteins at the T cell surface induces anergy or apoptosis (15, 17–19). Gal-3 induces anergy of CD8 T cells by distancing the T cell receptor (TCR) from the CD8 molecule, and impairs NK cell activity by inhibiting the interaction of the activating receptor natural-killer group 2, member D (NKG2D) expressed on NK cells and the heavily *O*-glycosylated tumor-derived MHC class I chain-related protein (MIC) A (15, 20, 21).

In this context, γδ T lymphocytes, which highly express NKG2D and infiltrate in PDAC tissues, are of high interest (22–25). In this study, we focused on Vδ2-expressing γδ T cells, which are specifically activated by pyrophosphate intermediates of the prokaryotic non-mevalonate pathway of cholesterol synthesis, and more importantly by dysregulated mevalonate-pathway metabolites of transformed eukaryotic cells (26, 27). Vδ2-expressing γδ T cells are a promising cell population for T cell-based immunotherapy due to their HLA-unrestricted target cell recognition and their enhanced cytotoxicity against PDAC cells after application of targeted biologicals such as bispecific antibodies (bsAb) (23, 28–30). Here, we were interested in a possible induction of anergy or apoptosis in Vδ2 γδ T cells by gal-3, which could explain the observed exhaustion of anti-tumor responses of Vδ2 γδ T cells against PDAC cells unless bsAb were applied (23, 30, 31).

## Materials and Methods

### Cohort and Ethic Statement

The Department of Transfusion Medicine of the University Hospital Schleswig-Holstein (UKSH) in Kiel, Germany, provided leukocyte concentrates from healthy adult blood donors. In addition, heparinized blood, serum samples and tumor tissue from PDAC patients were obtained from the Department of General and Thoracic Surgery (UKSH, Campus Kiel) and from the Surgery Department of the Community Hospital in Kiel distributed by the Biobank BMB-CC of the PopGen 2.0 Biobanking Network (P2N; UKSH, Campus Kiel) supervised by Dr. C. Röder (Institute for Experimental Cancer Research, Kiel, Germany). In total, 19 patients with histologically verified PDAC (stage pT2-3, pN0-2, L0-1, V0-1) were enrolled. Serum samples of 9 patients with histologically verified advanced ovarian cancer (FIGO-stage IIIA-IV) were obtained from the Department of Gynecology and Obstetrics of the UKSH in Kiel. Pathological features of all tissues were assessed according to WHO classification and UICC TNM staging. None of the patients had undergone chemo- or radiotherapy before this investigation. In accordance with the Declaration of Helsinki, written informed consent was obtained from all donors, and the research was approved by the relevant institutional review boards (Ethic Committee of the Medical Faculty of the CAU Kiel, code number: D405/10, D445/18, and A110/99).

### *Ex vivo* Isolation of Tumor-Infiltrating Lymphocytes and Tumor Cells

Tumor tissue of PDAC patients removed during surgery was dissected in the Institute of Pathology of the UKSH, Campus Kiel. Tumor tissues (1–2 cm^3^) were washed (in 10 cm dishes) with PBS to remove blood debris. Subsequently, the tumor tissues were minced into approximately 1 mm^3^ pieces and treated with components A, H, and R of the Tumor Dissociation Kit (Miltenyi Biotec, Bergisch Gladbach, Germany) for 1 h at 37°C in 5 mL PBS in a Gentle MACS (Miltenyi Biotec). Digested cell suspension was then passed through a 100 μm cell strainer (Falcon, BD Biosciences), visually controlled by light microscopy and centrifuged at 481 × g for 5 min. Tumor cells as well as tumor-infiltrating cells (TIL) were isolated by Ficoll-Hypaque (Biochrom, Berlin, Germany) density gradient centrifugation. The purity of the cells was determined by staining as described in the flow cytometry section. Cells were cultured in RPMI 1640 supplemented with 2 mM L-glutamine, 25 mM HEPES, 100 U/mL penicillin, 100 μg/mL streptomycin, 10% fetal bovine serum (FBS, Thermo Fisher Scientific, Karlsruhe, Germany) [complete medium].

*Ex vivo* isolated tumor cells and autologous TIL were characterized phenotypically and functionally as described under flow cytometry and functional assay section.

### Separation of PBMC and T Cells and Generation of Short-Term Activated T Cell Lines

Peripheral blood mononuclear cells (PBMC) were isolated from leukocyte concentrates or heparinized blood from PDAC patients by Ficoll-Hypaque (Biochrom) density gradient centrifugation. CD4 αβ T cells, CD8 αβ T cells and γδ T cells were positively separated from freshly isolated PBMC by using the magnetic cell separation system (Miltenyi Biotec). To isolate CD4 and CD8 T cells, cells were labeled directly with specific microbead-coupled mAbs (CD4 and CD8 MicroBeads, Miltenyi Biotec). To isolate γδ T cells, an indirect two-step process (anti-TCR γδ micro-Bead Kit, Miltenyi Biotec) consisting of labeling the γδ T cells with a specific hapten-coupled mAb followed by staining the cells with FITC-labeled anti-hapten microbeads were applied. The purity of the cells was >98% after their magnetic separation.

To expand short-term activated CD4 or CD8 αβ T cell lines or γδ T cell lines, 10^6^ cells/mL were cultured in 24-well plates in complete medium with 50 IU/mL rIL-2 (Novartis, Basel, Switzerland) and stimulated with Activation/Expander Beads (Miltenyi Biotec) with a one bead/one cell ratio for 3–4 days for αβ T cells and 5–10 days for γδ T cells. The beads were coated with 10 μg/mL each anti-CD3 and anti-CD28 mAbs and 0.5 μg/mL anti-CD2 mAb overnight and used as T cell receptor (TCR) stimulus. Alternatively, γδ T cell lines were expanded by stimulation of PBMC with 2.5 μM aminobisphosphonate (n-BP) zoledronic acid (Novartis), which induces a selective outgrowth of Vγ9 Vδ2-expressing γδ T cells. Since resting, initially stimulated γδ T cells produced only very low amounts of IL-2, 50 IU/mL rIL-2 was added every two day. After 2 weeks, Vγ9Vδ2-expressing γδ T cell lines had a purity of 60–99% and were labeled with anti-TCRαβ mAb clone IP26 (BioLegend, San Diego, CA) and subjected to magnetic separation in order to deplete remaining αβ T cells. After αβ T cell depletion, Vγ9 Vδ2 γδ T cell lines had a purity of 98–99%.

### Established Tumor Cell Lines and Cell Culture Conditions

Human PDAC cell lines Panc-1, PancTu-I, BxPC3, MiaPaCa-2, Capan-2 cells derived from primary tumors as well as Panc89 and Colo357 cells derived from a lymph node metastasis were cultured in complete medium under regular conditions (5% CO_2_, humidified, 37°C) (32). The PDAC cell lines were kindly provided by Dr. C. Röder and Prof. Dr. A. Trauzold, Institute for Experimental Cancer Research, Kiel, Germany. 0.05% trypsin/0.02% EDTA was used to detach adherent PDAC cell lines from flasks. Absence of mycoplasma was routinely confirmed by RT-PCR (Venor® GEM classic, Minerva Biolabs GmbH, Germany) and genotype by short tandem repeat analysis.

### Flow Cytometry

In total, 1–2 × 10^6^ PBMC and *ex vivo* isolated TIL were stained by multi-color flow cytometry approach to distinguish between diverse T cell subpopulations within different CD45^+^ leukocyte populations. Directly conjugated mAbs included PerCP-labeled anti-CD45 clone 2D1, PE-Cy7-labeled anti-pan TCRγδ clone 11F2 (both BD Biosciences, Heidelberg), AF700-labeled anti-CD3 clone SK7, BV510-labeled anti-CD4 clone OKT4, APC-Cy7-labeled anti-CD8 clone SK1 (all three, BioLegend), VioBlue-labeled anti-Vδ1 clone REA173 (Miltenyi Biotec), PE-labeled anti-Vδ2 clone B6 (BD Biosciences), and corresponding isotype controls (BD Biosciences or BioLegend).

To determine purity and expression of tumor-associated antigens such as epithelial cell adhesion molecule (EpCAM) and human epidermal growth factor receptor (HER)-2, 2 × 10^5^ *ex vivo* isolated tumor cells derived from tumor tissues were stained with mAbs as follows: PerCP-labeled anti-CD45 clone 2D1 (BD Biosciences), PE-Vio770-labeled anti-HER-2 clone 24D2 and APC-labeled anti-EpCAM clone REA-125 (both from Miltenyi Biotec) followed by intracellular staining with FITC-labeled anti-pan-Cytokeratin mAb clone CK3-6H5 (Miltenyi Biotec). All *ex vivo* isolated tumor cells were pan-Cytokeratin^+^, EpCAM^+^ and HER-2^+^, but did not express CD45. Additionally, TIL, *ex vivo* isolated tumor cells and established PDAC cell lines were also intracellularly stained with AF647-conjugated anti-gal-3 (clone M3/38) or an appropriate isotype control (an AF647-conjugated rat IgG2a mAb). Briefly, for the intracellular staining, 2–5 × 10^5^ cells were washed with staining buffer, fixed and permeabilized with the Cytofix/Cytoperm kit (BD Biosciences) for 20 min following the procedures outlined by the manufacturer. Thereafter, cells were washed twice with Perm/Wash by centrifugation and stained with fluorochrome-conjugated anti-gal-3 mAb or isotype control for 30 min, washed and measured.

All samples were analyzed on a FACS Calibur and a LSR-Fortessa flow cytometer (both from BD Biosciences) using CellQuestPro, Diva 8, or FlowJo software.

### Cell Death Analysis by Flow Cytometry

Cell death analysis of γδ T cells was performed by combined annexin-V FITC and propidium iodide (PI) staining. Briefly, 10^6^/mL short-term activated γδ T cells were treated with medium or different concentrations (0.1 and 1 μg/mL) of gal-3 or galectin (gal)-9 (both from BioLegend) in complete medium in 24-well plates for 24 h. After incubation, cells were washed with annexin-V binding buffer (MabTag, Friesoythe, Germany) and stained with annexin-V FITC (1:10, MabTag) and PI (2 μg/mL, Serva; Heidelberg, Germany). After two washing steps, cells were analyzed by flow cytometry, and the proportion of viable (annexin-V^−^ PI^−^), early apoptotic cells (annexin-V^+^ PI^−^) and late apoptotic/necrotic cells (annexin-V^+^ PI^+^) was determined.

### RNA Interference

In total, 1.5 × 10^5^ PDAC cells were seeded in 12-well plates, and incubated for 24 h in cell culture medium without antibiotics. To downregulate gal-3 expression, the cells were transfected with 12 pmol gal-3 Stealth RNAi™ siRNA or non-targeting control pool (gal-3 sense; # 10620318, non-targeting gal-3 antisense; # 10620319, both Thermo Fisher Scientific) using 2 μL Lipofectamine® RNAiMAX reagent (Thermo Fisher Scientific) in 200 μL Opti-MEM medium for 10–20 min according to manufacturer's protocol. The optimal time point of downregulation was analyzed by flow cytometry and by Western blot. The lowest gal-3 expression was detected 72 h after transfection.

### Functional Cell Culture Assay

In total, 1.25 × 10^5^ PBMC of healthy donors or PDAC patients were plated in complete medium with 50 IU/mL rIL-2 in 96-round well-plates. γδ T cells within PBMC were selectively activated by 300 nM phosphorylated antigen (PAg) bromohydrinpyrophosphate (BrHPP, Innate Pharma, Marseille, France) or 2.5 μM n-BP zoledronic acid in the absence or presence of different concentrations (0.01–10 μg/mL in logarithmic steps) of gal-3 (BioLegend). IFN-γ was determined in the supernatant after 48 h using ELISA (Supplemental Methods and Supplemental Figure 2). After 6–7 days, γδ T cell proliferation was analyzed as described in the Cell proliferation assay section. Alternatively, 5–10 × 10^3^ established PDAC cells (wild type, control or gal-3 siRNA transfected cells) were plated in 96-well flat-bottom plates in complete medium. After 24 h, 2.5 × 10^5^ PBMC were added together with 50 IU/mL rIL-2 at an effector/target (E/T) ratio of 50:1 (PBMC/tumor cells) representing an effective E/T of 15:1–40:1 for CD3 T cells/tumor cells or of 1:4–10:1 for Vγ9Vδ2 T cells/tumor cells. The effective E/T ratio was determined by staining PBMC with anti-CD3, anti-Vγ9 and anti-Vδ2 mAb using flow cytometry. In several experiments, resting or short-term activated CD4 and CD8 positively isolated αβ T cells or γδ T cells were used as effector cells at an E/T ratio of 50:1 for resting cells or 5:1–40:1 for activated T cells. αβ T cells were stimulated with Activation/Expander Beads (Miltenyi Biotec) coated with 10 μg/mL each of anti-CD3 and anti-CD28 mAbs and 0.5 μg/mL anti-CD2 mAb as αβ TCR stimulus or with 1 μg/mL bsAb [HER2xCD3], which targets HER-2 expressed on PDAC cells to CD3-expressing T cells (23, 30). γδ T cells were cultured with 50 IU/mL IL-2 (for resting cells) and 12.5 IU/mL (for activated cells), and were stimulated by 300 nM PAg BrHPP or 2.5 μM zoledronic acid or with the tribody [(HER2)_2_ xVγ9], which targets HER2-expressing PDAC cells to Vγ9-expressing γδ T cells (23, 30). As control, T cells were cultured and stimulated in the absence of PDAC cells. γδ T cell proliferation was analyzed after 6–7 days and αβ T cell proliferation after 3–4 days.

To analyze cell-cell contact dependency, 2 × 10^5^ PancTu-I cells plated in 24-well plates were cocultivated with unstimulated or with PAg-stimulated short-term activated Vγ9Vδ2 γδ T cells in the presence of 12.5 IU/mL IL-2 at an E/T ratio 40:1 separated or not by a membrane with 0.4 μm pores. As control, Vγ9Vδ2 γδ T cells were cultured alone. After 24 h, gal-3 was measured in the cell culture supernatant as described in the ELISA section.

For blocking assays, 2.5 × 10^5^ PBMC were pre-incubated in 110 μL of 10–20 μg/mL of neutralizing antibodies including anti-CD7 (clone M-T701), anti-CD11a (clone HI111), anti-CD29 (clone Mab 13), anti-CD49c (clone C3 II.I) (all from BD Biosciences) and anti-CD45 (clone HI 30, from BioLegend) as well as appropriate isotypes mIgG1 (MOPC-21, BioLegend), and rIgG2a (RTK2758, BioLegend). Alternatively, cells were pretreated with a combination of 10 μg/mL anti-CD29 mAb and 10 μg/mL anti-CD49c mAb or appropriate controls. After 2 h of incubation, 50 μL of pretreated PBMC were transferred to 5 × 10^3^ PancTu-I cells adhered overnight and stimulated with 2.5 μM zoledronic acid and 50 IU/mL of rIL-2. γδ T cell proliferation was analyzed after 7 days.

For autologous assay system, 5 × 10^3^ *ex vivo* isolated tumor cells of PDAC patients were plated in 96-well flat-bottom plates in complete medium. After 24 h, 2.5 × 10^5^ autologous PBMC were added together with 50 IU/mL rIL-2 at an effector/target (E/T) ratio of 50:1 (PBMC/tumor cells) representing an effective E/T of 1:1 or 0.5:1 (Vγ9Vδ2 T cells/tumor cells). For coculturing of *ex vivo* isolated tumor cells of PDAC patients with autologous TIL, we used a lower E/T ratio. This reduced E/T ratio resulted from a low number of isolated TIL derived from 1 cm^3^ PDAC tissue and from a different distribution of the cells within the tumor tissue of PDAC patients. While 40–60% expressed pan-Cytokeratin (detected as tumor cells) and 10–30% CD45 (detected as leukocytes), residual cells are tumor-associated cells, which are part of the TIL population after Ficoll-Hypaque density centrifugation of digested tumor tissue. 1.5 × 10^5^ *ex vivo* isolated tumor cells of PDAC patients were plated in 96-well flat-bottom plates in complete medium. After 24 h, 1.5 × 10^3^ autologous TIL were added together with 50 IU/mL rIL-2 at an effector/target (E/T) ratio of 1:4 (TIL/tumor cells) representing an effective E/T of 1:40 or 1:400 (Vγ9Vδ2 T cells/tumor cells).

Functional read out systems included a proliferation assay and measurement of gal-3 release as described as follows.

### Cell Proliferation Assay

Proliferation was determined by measuring the absolute cell number of viable CD3^+^ γδ or αβ T cells with a flow cytometric method termed standard cell dilution assay (SCDA) after 6–8 days of culture. SCDA has been reported as a precise method to specifically quantify any subset of phenotypically definable, viable cells in heterogeneous populations (33). Briefly, co-cultured T cells and tumor cells from 96-well round-bottom plates were washed and stained with fluorescein isothiocyanate (FITC)-labeled anti-CD3 mAbs (BD Biosciences, Heidelberg, Germany) or AF488-conjugated anti-Vγ9 mAb clone 7A5 (34). After one washing step, cells were resuspended in 100 μL sample buffer containing a defined number (10^4^) of APC-labeled fixed standard cells and 0.2 μg/mL PI. The standard cells were purified T cells that had been stained with APC-labeled anti-HLA class I mAb clone W6/32 and anti-TCRαβ mAb clone IP26, and fixed in 1% paraformaldehyde. The analysis on a flow cytometer allowed us to simultaneously measure the expansion of viable CD3 or Vγ9 γδ T cells (FITC^+^ or AF488^+^ PI^−^ APC^−^) and standard cells (FITC^−^ or AF488^−^ PI^+^ APC^+^). Based on the known number of standard cells, the absolute number of viable CD3 T cells or Vγ9 γδ T cells in a given microculture well could be determined as follows: T cell subset ratio = relative proportion of FITC^+^ or AF488^+^ PI^−^ APC^−^ stained T cells/relative proportion of FITC^−^ or AF488^−^ PI^+^ APC^+^ standard cells. The absolute number of FITC^+^ or AF488^+^-stained T cells can be determined by multiplying the T cell subset ratio with the number of standard cells per sample (10^4^ per 100 μL), since there is a linear correlation between the T cell subset ratio and the absolute number of FITC^+^ or AF488^+^-stained T cells as previously described (33). All samples were analyzed on a FACS Calibur (BD Biosciences) using CellQuestPro (including Batch-Setup function). Data were transferred to MS-Excel for further analysis.

### Enzyme-Linked Immunosorbent Assay

To quantify gal-3 released by PDAC cells (established cell lines or *ex vivo* isolated tumor cells) or T cells alone or after coculture of the different cell subsets, supernatants were collected after different incubation times (24–96 h) and stored at −20°C until use. Additionally, gal-3 was determined within serum samples of PDAC patients. Gal-3 was measured by sandwich DuoSet ELISA kit (# DY1154 from R&D System, Wiesbaden, Germany) in duplicates following the procedures outlined by the manufacturer.

### ^51^Cr-Release Assay

Control or gal-3 siRNA transfected PDAC cells were labeled with 50 μCi sodium ^51^Cr and 5 × 10^3^ cells were used as targets in a standard 4 h ^51^Cr release assay with titrated numbers of short-term activated γδ T cells as effectors at an E/T ratio of 50:1, 25:1, 12.5:1, and 6.25:1. Cells were (co)cultured in medium or stimulated with the bsAb [(HER2)_2_xVγ9] for 4 h. Supernatants were measured in a MicroBeta Trilux β-counter (PerkinElmer, Hamburg, Germany). Specific lysis was calculated as [(cpm test–cpm spontaneous)/(cpm max–cpm spontaneous)] × 100, where spontaneous release was determined in medium only and maximal release was determined in Triton-X-100 lysed target cells. Spontaneous release did not exceed 15% of the maximal release.

### Imaging Flow Cytometry

The localization of proteins and their colocalization with other proteins was quantified by an ImageStream®X Mark II (Merck Millipore, Burlington, MA, USA). This device combines flow cytometry with microscopy by using a camera (with 405, 488, 562, 658, and 732 nm lasers) which takes high-resolution images of each cell in up to six fluorescence channels and analyzes up to 5,000 cells/s. To investigate the localization of gal-3 in PDAC cells, 10^6^ PDAC cells were stained with 10 μg/mL anti-gal-3 mAb clone Gal397 (BioLegend) followed by 10 μg/mL AF488-conjugated goat anti-mouse secondary Ab (Thermo Fisher Scientific) according to the protocol for intracellular staining (see section flow cytometry). These images were then analyzed using the IDEAS® image analysis software.

### Analysis of Synapse Formation Between Tumor Cells and T Cells

To investigate the effect of synapse formation on the localization of gal-3, PancTu-I cells were pelleted in 15 mL Eppendorf tubes and an equal number of short-term activated Vγ9Vδ2 γδ T cells as effector cells were added. After 1, 3, 5, 10, 20, or 45 min, the cell conjugates were pelleted, fixed and permeabilized with Cytofix/Cytoperm kit (BD Biosciences), and transferred to 96-well plates for staining. Thereafter, cell conjugates were stained with 10 μg/mL anti-gal-3 Ab clone M3/38 (BioLegend) followed by staining with 10 μg/mL AF555-conjugated goat anti-rat secondary Ab according to the protocol for intracellular staining. After a further washing step, cell conjugates were stained with an Ab mixture of 0.6 μg/mL APC-conjugated anti-EpCAM mAb clone REA-125 (Miltenyi) for tumor cells, 5 μg/mL BV421-conjugated anti-CD3 mAb clone UCHT-1 (BD Biosciences) for T cells, and CruzFluor-488-conjugated phalloidin (1:5000, Santa Cruz, Heidelberg) for actin-filament staining which is condensed at the immunological synapse after tumor-T cell interaction. After washing, the cells were measured on ImageStream®X Mark II (Merck Millipore) and conjugates were analyzed using the IDEAS® image analysis software. Firstly, tumor-T cell conjugates were identified *via* EpCAM, CD3, and phalloidin expression. To define the cell periphery, a peripheral mask was then generated based on the EpCAM signal, which marks the surface of the tumor cell. For this purpose, a mask was defined with the *Dilate*-function of the software that is one pixel larger than the mask of the EpCAM signal. In addition, a mask was defined with the Erode-function of the software that is six pixels smaller than the mask of the EpCAM signal. Subtraction of the *Erode*-generated mask from the *Dilate*-generated mask provided a ring-shaped peripheral mask. The peripheral mask protrudes one pixel beyond the cell and six pixels into the cell that allows the determination of the gal-3 signal in the cell periphery.

### Statistical Analysis

**Figure 1:**
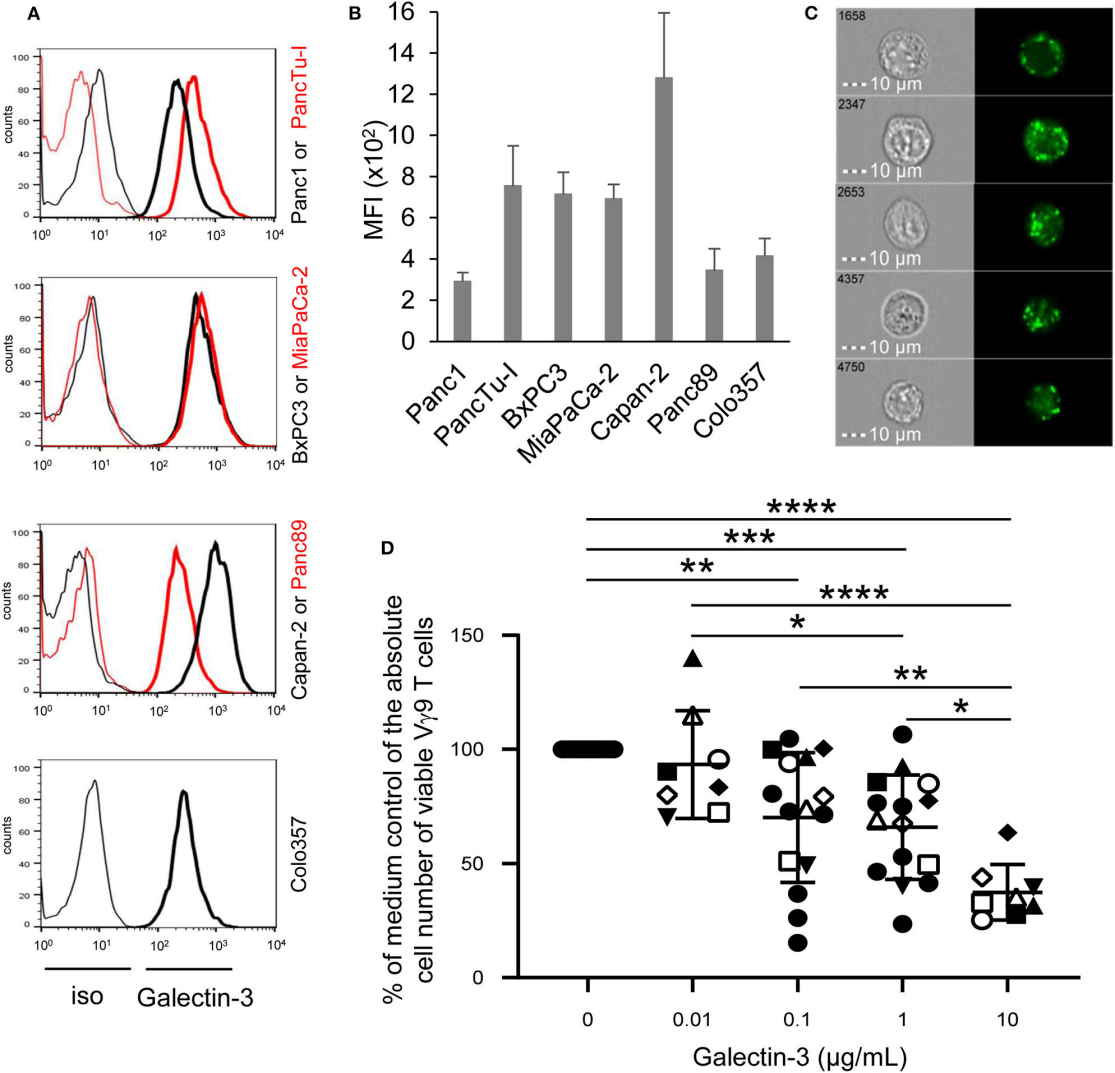
Intracellular galectin-3 expression in PDAC cells, and effect of recombinant galectin-3 on γδ T cell proliferation. **(A)** Histograms depicted are representative results of indicated PDAC cells. Thin and bold lines represent isotype control and gal-3 expression (clone M3/38), respectively. **(B)** Median fluorescence intensity (MFI) ± SD (*n* = 3, duplicates) of gal-3 expression corrected by the MFI of the isotype control is shown for the indicated PDAC cell lines measured by FACS Calibur. **(C)** PancTu-I cells were labeled with anti-gal-3 mAb (clone Gal397) and analyzed on the ImageStream® X Mark II. Five representative cells out of 5 × 10^3^ recorded cells are shown with bright field image (left side) and fluorescence image of gal-3 (right side). Scale bars represent 10 μm. **(D)** 1.25 × 10^5^ PBMC of paired healthy donors (*n* = 4, different closed symbols), additional healthy donors (*n* = 7, two conc. of gal-3, closed circles) and paired PDAC patients (*n* = 4, open symbols) were stimulated with 300 nM BrHPP with the different indicated concentrations of rgal-3 and 50 IU/mL rIL-2. After 6–7 days, the absolute cell number was determined as a percentage of the medium control of the Vγ9 γδ T cells using SCDA. The mean ± SD of duplicates is shown. Statistical comparison of non-matched samples was carried out parametrically by using one-way ANOVA followed by Tukey's multiple comparison test. Significances are shown as *P*-value; *P < 0.05, **P < 0.01, ***P < 0.001, ****P < 0.0001.

**Figure 2:**
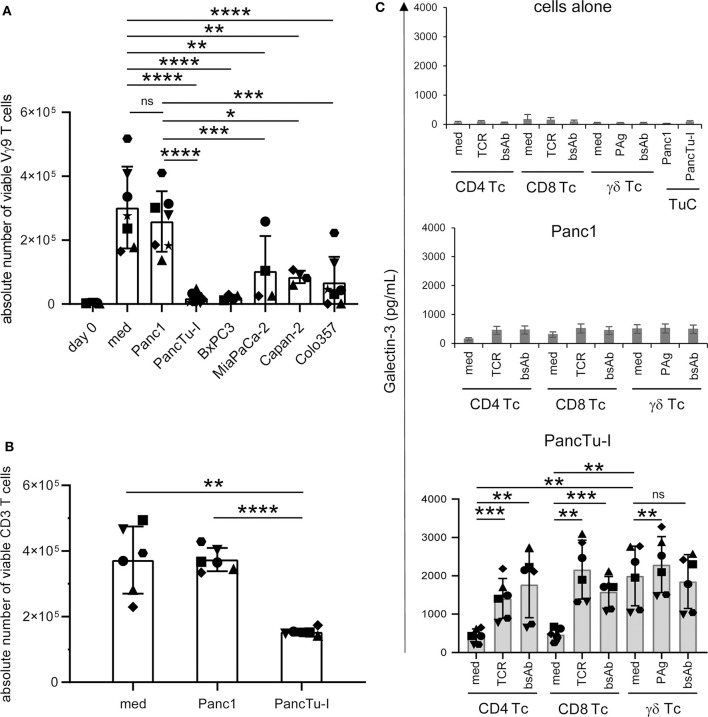
Coculture of PDAC cells with PBMC induces galectin-3 release and inhibition of γδ T cell proliferation. **(A–C)** In total, 5 × 10^3^ of the indicated PDAC cells were plated in complete medium. After 24 h **(A,B)** 2.5 × 10^5^ PBMC (*n* = 4–7) or **(C)** 2 × 10^5^ freshly isolated CD4 or CD8 αβ T cells or γδ T cells (Tc) (*n* = 6) were added or cultured alone (medium). Cells were stimulated **(A)** with 2.5 μM zoledronic acid in the presence of 50 IU/mL rIL-2, **(B)** with Activation/Expander Beads or **(C)** with Activation/Expander Beads (TCR), 300 nM BrHPP (PAg) plus rIL-2 or 1 μg/mL bsAb [HER2xCD3] for αβ T cells and [(HER2)_2_xVγ9] plus rIL-2 for γδ T cells. **(A,B)** Vγ9 γδ T cell proliferation was determined after 6–7 days and αβ T cell proliferation after 3–4 days by SCDA. The means ± SD of duplicates are shown. **(C)** Cell culture supernatants were collected after 72 h and released gal-3 was determined by ELISA. The bars represent the mean ± SD (*n* = 6), determined in duplicates. Statistical comparison of **(A)** non-matched samples was carried out parametrically by using one-way ANOVA followed by Tukey's multiple comparison test, and of **(B,C)** matched samples also parametrically by using paired, two-tailed *t*-test. *P*-value; *P < 0.05, **P < 0.01, ***P < 0.001, ****P < 0.0001, and ns, non-significant.

**Figure 3:**
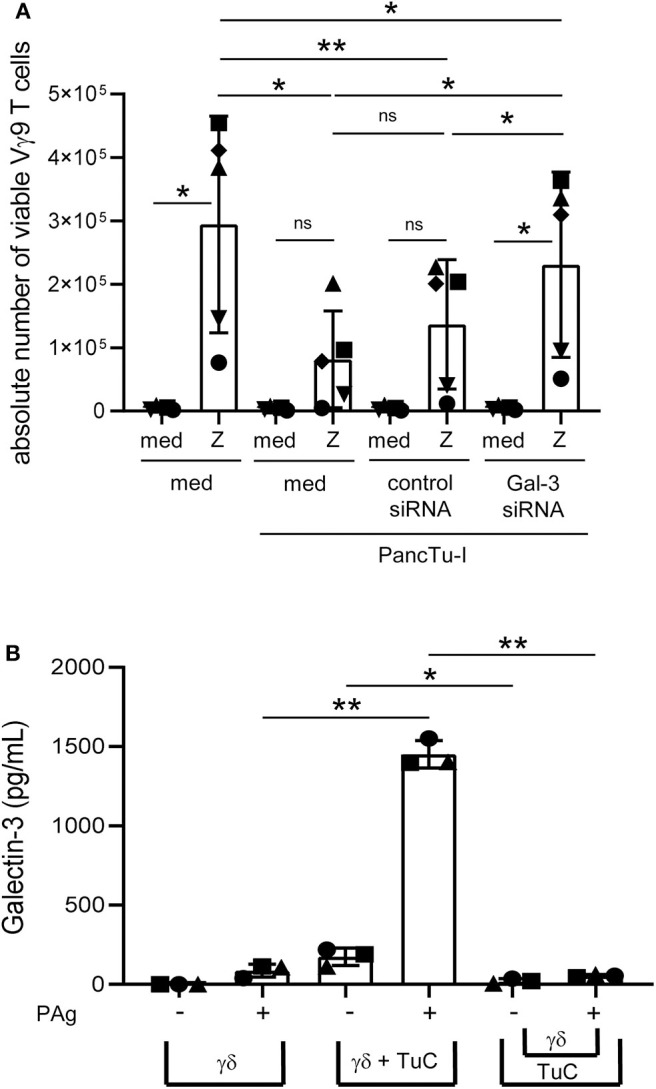
Galectin-3 knockdown in PancTu-I cells partially restores γδ T cell proliferation within PBMC, and gal-3 release in coculture is cell contact-dependent. **(A)** In total, 5 × 10^3^ PancTu-I cells left non-transfected or were transfected with either control siRNA or gal-3 siRNA and cultured in complete medium 72 h after transfection. After 24 h culture of PDAC cells, 2.5 × 10^5^ PBMC were added (E/T 50:1) or cultured alone. After addition of 50 IU/mL rIL-2, cells were left unstimulated (med) or stimulated with 2.5 μM zoledronic acid (Z). After 6 days, the absolute cell number of the Vγ9 γδ T cells was determined using SCDA. The mean ± SD of duplicates from 5 donors are shown. **(B)** Short-term activated Vγ9Vδ2 T cells were cultured with 50 IU/mL rIL-2 alone and directly cocultured with 2 x 10^4^ PancTu-I cells or indirectly separated by a semipermeable membrane with 0.4 μm pores of a transwell insert in 24-well plates at an E/T ratio of 40:1. Cells were left untreated or stimulated with PAg. After 24 h, gal-3 was measured in the cell culture supernatant using ELISA. The means ± SD of 3 donors are shown. **(A,B)** Statistical comparison of matched samples was carried out parametrically by using paired, two-tailed *t*-test. *P*-value; *P < 0.05, **P < 0.01, ns, not significant.

**Figure 4:**
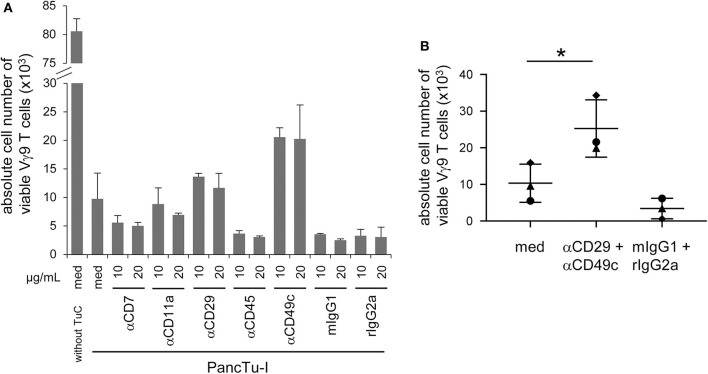
CD49c/CD29 play a role in the galectin-3 mediated inhibition of γδ T cell proliferation within PBMC. **(A,B)** In total, 5 × 10^3^ PancTu-I cells were seeded for 24 h. 2.5 × 10^5^ PBMC/well were pre-incubated with **(A)** the indicated concentrations of the displayed antibodies or appropriate isotype controls or **(B)** with 10 μg/mL anti-CD29 mAb together with 10 μg/mL anti-CD49c mAb or appropriated isotype controls for 2 h. **(A,B)** Thereafter, PancTu-I cells were transferred and stimulated with 2.5 μM zoledronic acid and 50 IU/mL rIL- 2. After 7 days, the absolute cell number of Vγ9 γδ T cells was determined using SCDA. **(A)** The means ± SD of duplicates of 2 donors are shown. **(B)** Each point represents an individual donor. Statistical comparison of matched samples was carried out parametrically by using paired, two-tailed *t*-test. *P*-value; *P < 0.05.

**Figure 5:**
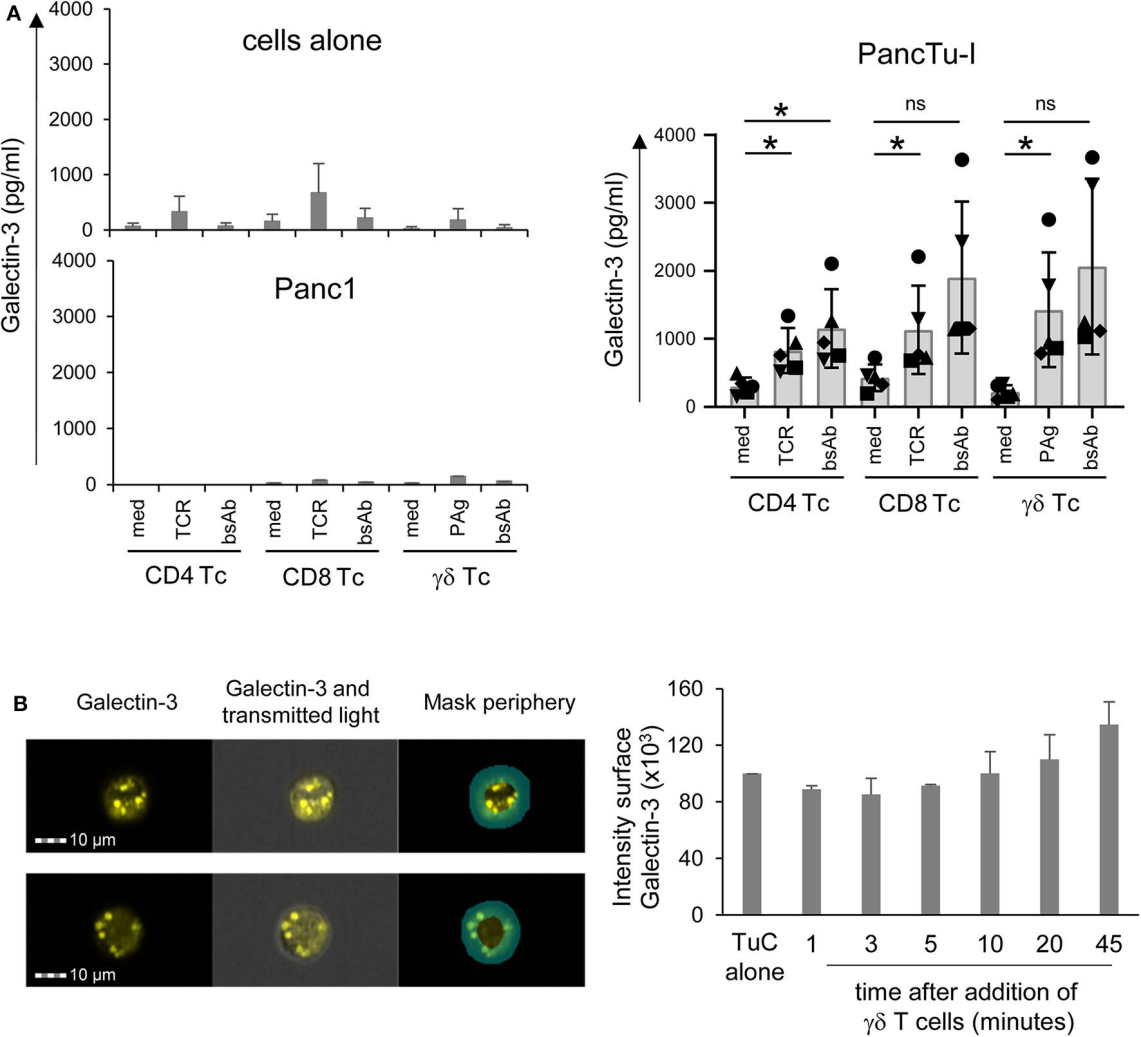
Coculture of PancTu-I cells with activated γδ T cells increases galectin-3 release and its localization in the tumor cell periphery. **(A)** In total, 5 × 10^3^ indicated PDAC cells were plated in complete medium. After 24 h, 2 × 10^5^ short-term activated CD4 or CD8 αβ T cells or γδ T cells (Tc) (*n* = 6) were added or cultured alone (cells alone). Cells were stimulated with Activation/Expander Beads (TCR), 300 nM BrHPP (PAg) plus 12.5 IU/mL rIL-2 or 1 μg/mL bsAb [HER2xCD3] for αβ T cells and [(HER2)_2_xVγ9] plus 12.5 IU/mL rIL-2 for γδ T cells. Cell culture supernatants were collected after 72 h and gal-3 was determined by ELISA. The means ± SD of duplicates are shown. Statistical comparison was carried out parametrically by using paired, two-tailed *t*-test. *P*-value; *P < 0.05. As samples of CD8 and γδ T cells cultured in medium in comparison to bsAb did not follow a normal distribution, a Wilcoxon non-parametric, matched-pairs signed rank test was applied, which revealed no significance (ns, non-significant). **(B)** PancTu-I cells alone or cocultured with short-term activated Vγ9 γδ T cells for the indicated time. Thereafter, cells were stained with anti-gal-3 mAb (clone Gal397) followed by secondary goat-anti-mouse Ab and anti-EpCAM mAb (clone REA-125) for tumor cells, anti-CD3 (clone UCHT-1) for T cells and phalloidin for actin-filament staining and analyzed on the ImageStream® X Mark II. The fluorescence image of gal-3 plus overlays with transmitted light image and peripheral mask of a representative PancTu-I cell with gal-3 expression predominately in the center or periphery is shown. In addition, the mean ± SD of the intensity of gal-3 expression in the periphery of PancTu-I cells after addition of γδ T cells (*n* =3) are presented.

Shapiro-Wilk normality test was applied to analyze the normal distribution assumption. The statistical analysis was assessed by using Graph Pad Prism (Graph Pad Software, Inc., La Jolla, CA, USA). The assumption of normal distribution was given in all samples [Figures 1–**4**, **7**, **8A,B** (right panel)] with the exception of parts of **Figures 5A**, **7** (γδ T cells), **8B** (left panel) as indicated in the appropriate figure legend. For parametric data of non-matched datasets, a one-way ANOVA followed by Tukey's multiple comparison test was carried out. For parametric data of matched datasets, a paired, two-tailed *t*-test was used. Non-parametric data of matched datasets were analyzed by Wilcoxon matched-pairs signed rank test. All statistical tests were two-sided and the level of significance was set at α ≤ 5%. Tests are indicated in the figure legends where appropriate.

## Results

### Galectin-3 as a Tumor-Suppressive Mediator of (γδ) T Cell Proliferation

Recently, we and others demonstrated that γδ T cells infiltrate the tumoral ducts of PDAC tissue (23, 24, 35). A possible reason for the weak anti-tumor response of tumor-infiltrating γδ T cells may be due to gal-3, which is described to be expressed by PDAC cells and to contribute to tumor-mediated immune suppression (13, 15). Regarding the expression of gal-3 in tumor cells, we examined PDAC cells of different origin and differentiation grade. Gal-3 was expressed very weakly in Panc1 and Panc89 cell lines derived from primary tumors as well as in Colo357 cells derived from a lymph node metastasis. Interestingly, all other primary PDAC cells expressed intracellularly gal-3 to a higher extent than Panc1, Panc89, and Colo357 cells (Figures 1A,B). In order to obtain further information on possible functions of gal-3, the localization of gal-3 in these PDAC cells was analyzed in more detail. PancTu-I cells which showed a high expression of gal-3 in comparison to Panc1 cells were labeled intracellularly with anti-gal-3 mAb and analyzed on an ImageStream® X Mark II. A clear cytoplasmic, vesicular localization of gal-3 was observed in all PDAC cells, also in Panc1 cells but to a much lesser extent (Figure 1C, data not shown). A partial colocalization of gal-3 with vesicular marker proteins such as the lysosomal membrane-associated proteins CD107a (LAMP-1) and CD63 (LAMP-3) as well as vesicle synaptosome-associated protein receptor Vti1b (expressed on vesicles of the trans-golgi network or late endosomes), respectively, was observed. The expression was found only in a small fraction of analyzed cells. However, no colocalization was found with the recycling endosomes-associated protein Rab11 (Supplemental Figure 1). This is in line with the described non-classical secretory pathways for gal-3. Interestingly, CD4 and CD8 TCRαβ T cells and TCRγδ T cells also showed a vesicular expression of gal-3 (data not shown).

To examine whether recombinant gal-3 has an influence on the proliferation of γδ T cells, PBMC of healthy donors and PDAC patients were stimulated with PAg BrHPP and IL-2 in the presence of different concentrations of recombinant gal-3. Whereas, BrHPP and IL-2 induced a selective outgrowth of γδ T cells within PBMC, the addition of increasing concentrations of gal-3 to stimulated γδ T cells significantly reduced their proliferation (Figure 1D). While the addition of 0.01 μg/mL gal-3 had no impact on γδ T cell proliferation, 0.1 and 1 μg/mL significantly impaired Vγ9Vδ2 T cell proliferation, respectively. In addition, 10 μg/mL gal-3 reduced the proliferation up to 60% compared to the medium control, suggesting a direct inhibitory effect on the proliferative capacity of γδ T cells. In contrast, the IFN-γ release after BrHPP and IL-2 stimulation was not significantly influenced in the presence of different gal-3 concentrations (Supplemental Figure 2).

The stimulation of PBMC with zoledronic acid induced an average 52-fold increase of the absolute cell number of viable γδ T cells within PBMC (medium) compared to day 0 (Figure 2A). Interestingly, coculturing these PBMC with weak gal-3 expressing Panc1 cells just leads to a minimal decrease of γδ T cell proliferation. Those primary PDAC cells, which expressed gal-3 to a higher extent, significantly suppressed γδ T cell proliferation during coculture (Figure 2A). Comparable results were obtained by stimulating all CD3-expressing T cells *via* their TCR. The TCR-induced CD3 T cell proliferation was not influenced by the coculture with Panc1 cells comparable to the stimulation without PDAC cells (medium), while the coculture with highly gal-3-expressing PancTu-I cells significantly reduced their proliferation (Figure 2B). Comparable results were obtained with other PDAC cells such as Capan-2 and BxPC3 both expressing gal-3 to a higher extent compared to Panc1 cells (data not shown). As already described, the suppression of γδ T cell proliferation in the presence of Colo357 cells is most likely due to an enhanced expression of cyclooxygenase-2 in Colo357 cells which induce prostagladin E2-mediated suppression (31, 36).

When PDAC cells or isolated T cells were cultured separately or T cells were stimulated *via* TCR or by bsAb, the release of gal-3 was weak, respectively (Figure 2C, upper panel, cells alone). Determination of gal-3 release in supernatants after 24, 48, and 72 h revealed the highest gal-3 release after 72 h (Figure 2C). Furthermore, we observed a slight increase of gal-3 secretion after coculturing the T cells with Panc1 cells (Figure 2C, middle panel). Interestingly, the gal-3 release significantly increased after coculture of isolated γδ T cells with PancTu-I cells compared to coculture with αβ T cells (Figure 2C, lower panel, medium). After polyclonal stimulation of T cells *via* the TCR or by bsAb, an enhanced gal-3 secretion was observed in the presence of PancTu-I cells (Figure 2C, lower panel, TCR, bsAb).

Taken together, gal-3 was expressed in PDAC cells and T cells, but was released only in small amounts by either cell population. However, large amounts of gal-3 were released during coculture of T cells together with highly gal-3 expressing PDAC cells such as PancTu-I cells. PDAC cells with a high amount of gal-3 release as well as soluble recombinant gal-3 inhibited the proliferation of T cells.

### Galectin-3 Released From Tumor Cells Inhibits γδ T Cell Proliferation

The release of gal-3 is drastically enhanced in the coculture of T cells and PancTu-I cells (Figure 2C). To examine whether the released gal-3 is responsible for the inhibition of T cell proliferation, gal-3 was knocked down by siRNA in PancTu-I cells. The functionality of different gal-3 siRNAs in PancTu-I cells was investigated on the basis of different applied concentrations and at the optimal time point using flow cytometry and Western blot analyses (Supplemental Figure 3). After 72 h, a knockdown of 85–90% of gal-3 expression could be shown after treatment with a concentration of 10–25 nM gal-3 siRNA in PancTu-I cells compared to control siRNA transfected cells (Supplemental Figure 3). Having defined the appropriate conditions, non-transfected and control or gal-3 siRNA transfected PancTu-I cells were cocultured with PBMC. After 6 days of coculture, a vigorous selective outgrowth of Vγ9 γδ T cells after stimulation with zoledronic acid compared to the control was observed by measuring absolute cell number of viable Vγ9 γδ T cells (Figure 3A). Because PBMCs from different donors contain varying numbers of Vγ9 Vδ2 γδ T cells, additionally, the x-fold increase of Vγ9 γδ T cells is presented (Supplemental Figure 4A). Non-transfected PancTu-I cells significantly inhibited the selective Vγ9 γδ T cell proliferation, while the coculture of gal-3 siRNA transfected PancTu-I cells partially, but significantly, restored the Vγ9 γδ T cell proliferation compared to the coculture with control siRNA transfected PancTu-I cells (Figure 3A). Similar results were obtained by applying BrHPP instead of zoledronic acid for stimulation of γδ T cell proliferation (Supplemental Figure 4B).

In contrast, Vγ9 γδ T cell proliferation after BrHPP or zoledronic acid stimulation of PBMC cocultured with non-transfected or control siRNA transfected Panc1 cells (with low gal-3 expression) did not differ from coculture with gal-3 siRNA transfected Panc1 cells (data not shown).

In order to induce a release of gal-3 by the PDAC cells during the coculture with T cells, soluble factors of T cells, a cell contact-dependent mechanism or both could play a role. To examine a possible cell-cell contact dependence of gal-3 release, PancTu-I cells were directly cocultured with short-term activated Vγ9Vδ2 γδ T cell lines or indirectly separated by a semipermeable membrane of a transwell insert for 24 h. When short-term activated Vγ9Vδ2 γδ T cell lines were cultured alone, only a small amount of gal-3 was released after stimulation. Release of gal-3 was significantly enhanced when cocultured with PancTu-I cells but not after separating the PDAC cells from the γδ T cell lines suggesting that the release of gal-3 is cell-cell contact-dependent (Figure 3B).

Similar effects were observed when using CD8 αβ T cell lines instead of γδ T cell lines or Capan-2 cells instead of PancTu-I cells (Supplemental Figure 5).

In sum, coculturing T cells and PDAC cells led to release of large amounts of gal-3. Knockdown of gal-3 in PDAC cells revealed that tumor cells were the source of gal-3 in coculture with T cells. The release of gal-3 by PDAC cells in coculture was dependent on cell contact with T cells, although it cannot be completely ruled out that next to direct cell contacts, soluble factors released by T cells may also be necessary to induce gal-3 release by the tumor cells.

### α3β1 Integrin (CD49c/CD29) Is Involved in the Inhibition of T Cell Proliferation

Gal-3 can bind separately to various proteins expressed on T cells such as CD7, CD11a (α-L integrin), CD29 (β1 integrin), CD45 and CD49c (α3 integrin) which could be responsible for the functional interaction of T cells and PDAC cells (1, 15, 17, 37). Therefore, PBMC were pretreated with relevant antibodies against CD7, CD11a, CD29, CD45, and CD49c or appropriate isotype controls at the indicated concentrations for 2 h, respectively. Thereafter, PBMC were stimulated with zoledronic acid combined with rIL-2 in the presence or absence of PancTu-I cells. After 6 days of culturing, γδ T cell proliferation was measured. As expected, the presence of PancTu-I cells inhibited the zoledronic acid-induced γδ T cell proliferation, and pretreatment with anti-CD7 and anti-CD45 mAbs further enhanced this inhibition (Figure 4A). Interestingly, only pretreatment of PBMC with anti-CD29 or anti-CD49c mAbs partially restored the proliferation of γδ T cells (Figure 4A). The partial restoration can be explained by the observation that antibody pretreated PBMC in the absence of cocultured PDAC cells already lead to a slight reduction of γδ T cell proliferation compared to medium only (data not shown). However, pretreatment with anti-CD49c mAb restored the γδ T cell proliferation after coculture with PDAC cells to a higher extent than pretreatment with anti-CD29 mAb suggesting that α3 integrin plays a greater role than β1 integrin (Figure 4A).

Interestingly, additional experiments that combined anti-CD29 and anti-CD49c mAbs also revealed only a partial but significant reconstitution of gal-3 mediated inhibition of γδ T cell proliferation (Figure 4B).

Taken together, gal-3-mediated Vγ9 γδ T cell inhibition by PDAC cells is partially and significantly restored by a combination of neutralizing anti-CD49c and anti-CD29 mAbs.

### Enhanced Galectin-3 Release of Gal-3-Expressing PDAC Cells Cocultured With γδ T Cells

In this study, we demonstrated that gal-3 release by PDAC cells is enhanced after coculture with resting T cells compared to culture of either cell population alone. While T lymphocytes including γδ T cells in tumor patients are often suggested to be in an activated state, we examined whether the release of gal-3 differs in short-term activated T cells (Figure 5A) in comparison to resting T cells (Figure 2C). Again, when PDAC cells or short-term activated T cells were cultured in medium alone or after T cell stimulation *via* TCR or by bsAb, the release of gal-3 was very low (Figure 5A, upper panel, cells alone). While the coculture of short-term activated T cells with Panc1 cells revealed no increase in gal-3 release after 72 h (Figure 5A, lower left panel), gal-3 release was enhanced after coculturing short-term activated T cells with PancTu-I cells as well as after their stimulation *via* the TCR- or by bsAb (Figure 5A, right panel). In sum, we obtained similar results with short-term activated T cells compared to resting T cells.

### Galectin-3 Is Relocalized Into the Cell Periphery of PDAC Cells in Coculture With T Cells

Hence, using ImageStream® X Mark II, we analyzed whether the release of gal-3 by PDAC cells was induced by the formation of an immunological synapse between interacting PDAC cells and T cells. To this end, short-term activated γδ T cells were cocultured with PancTu-I cells for 1–45 min. To distinguish between the two populations, T cells were stained with anti-CD3 mAb and PancTu-I cells with anti-EpCAM mAb. In order to demonstrate the formation of an immunological synapse, phalloidin was used as a marker for filamentous (F)-actin. In addition, gal-3 was stained to analyze whether gal-3 is released by PDAC cells when the immunological synapse is formed (Figure 5B). Regardless of the time point of γδ T cell addition, <1% of the PDAC and T cells formed conjugates (data not shown). This could indicate a “kiss and run mechanism” suggesting only a short contact between PDAC and T cells. Interestingly, a different gal-3 localization was observed depending on the coculture duration (Figure 5B). As described in the Materials and Methods section, a peripheral ring mask, which distinguishes between cell periphery and cell center, was used to analyze the intensity of gal-3 staining. Figure 5B shows two representative PancTu-I cells with considerable gal-3 expression in the cell center (Figure 5A, upper panel) as well as in the cell periphery (Figure 5A, lower panel). The intensity of gal-3 expression within the peripheral mask of PancTu-I cells (Tumor cells, TuC alone, *n* = 1) and of PancTu-I cells after the indicated times of coculture with Vγ9 γδ T cells (mean of *n* = 3 donors) was calculated (Figure 5B). The gal-3 expression decreased slightly in the cell periphery within the first 5 min after the addition of the γδ T cells which indicates a release of gal-3. Ten minutes after γδ T cell addition, a time-dependent increase of gal-3 expression intensity in the cell periphery was detected showing up to 30% enhanced gal-3 expression intensity after 45 min in comparison to PDAC cells culture in the absence of γδ T cells. These data indicate a possible relocalization of gal-3 from cell center toward cell periphery or cell surface.

Overall, these results indicate that PDAC cells transport gal-3 in vesicles to the cell surface after having direct cell contact with γδ T cells. Consequently, gal-3 is released and inhibits γδ T cell proliferation.

### Bispecific Antibody Enhanced γδ T Cell Cytotoxicity Against PDAC Cells Independent of Galectin-3 Knockdown

We next analyzed whether the γδ T cell cytotoxicity was influenced by gal-3 released by PDAC cells. Therefore, short-term activated γδ T cells were cocultured with Cr^51^-labeled control or gal-3 siRNA transfected PDAC cells at different effector/target ratio, and cytotoxicity was analyzed using Cr^51^-release assay.

**Figure 6:**
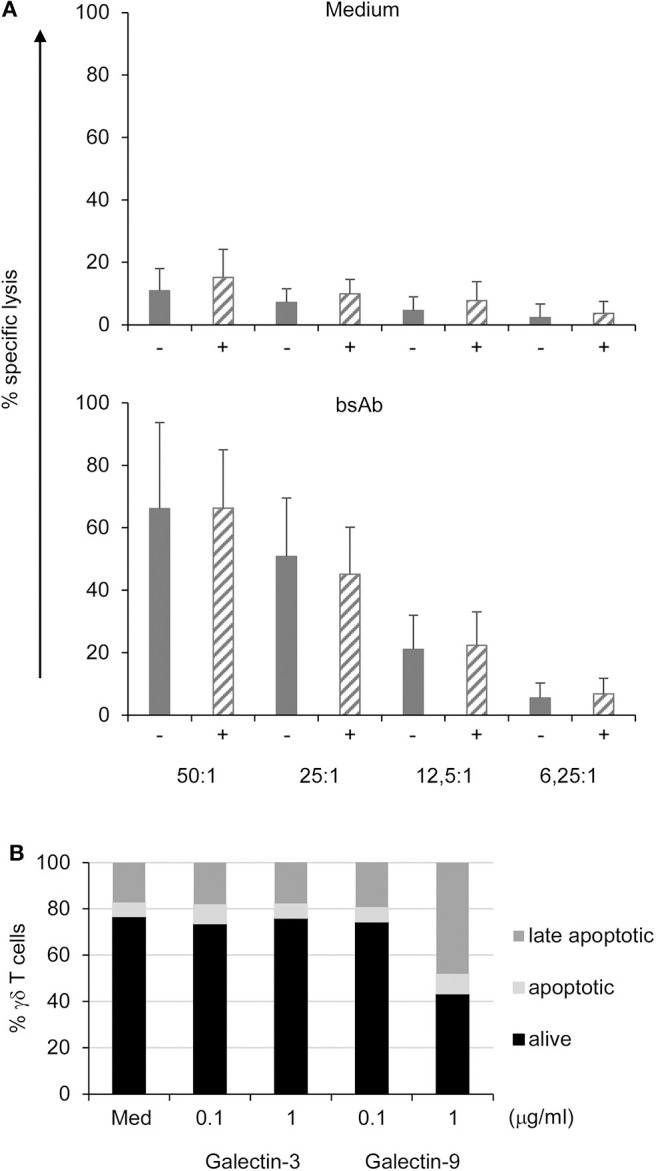
Galectin-3 knockdown does not influence γδ T cell-mediated cytotoxicity against PDAC cells, and does not induce cell death in γδ T cells. **(A)** In total, 5 × 10^3^ control (gray bars) or gal-3 siRNA transfected (striped bars) PancTu-I cells were labeled with ^51^Cr and used as targets in a standard ^51^Cr release assay. Short-term activated γδ T effector cells were titrated at the indicated E/T ratio and cultured in medium or stimulated with the bsAb [(HER2)_2_xVγ9]. Mean values of triplicates (SD < 10%) are calculated. Mean ± SD of 3 independent experiments with 3 different donors are shown. **(B)** 10^5^ short-term activated γδ T cells were treated with the indicated concentrations of gal-3 or gal-9 for 24 h. The cells were then labeled with annexin-V and propidium iodide (PI) and analyzed by flow cytometry. The proportion of alive (black bars, annexin-V^−^ PI^−^), early apoptotic (light gray bars, annexin-V^+^ PI^−^) and late apoptotic/necrotic (dark gray bars, annexin-V^+^ PI^+^) cells are shown as the mean value of 3 donors in 3 independent experiments. SD was <10%.

PancTu-I and Panc1 cells were almost resistant to γδ T cell-mediated lysis unless bsAb were added to the culture [Figure 6A and (38)]. The addition of bsAb significantly increased the γδ T cell cytotoxicity independent of the gal-3 knockdown (striped bars) in PDAC cells (Figure 6A) suggesting that gal-3 did not influence γδ T cell cytotoxicity against PDAC cells. In addition, CD107a-degranulation of γδ T cells was not significantly modulated by gal-3 knockdown (Supplemental Figure 6).

Moreover, γδ T cell cytotoxicity was not influenced by gal-3 in the presence or absence of bsAb. BsAb enhanced the cytotoxic activity as well as the release of granzyme A and B by γδ T cells (23, 24, 29). Similar to short-term activated γδ T cells, tumor-infiltrating γδ T cells expressed high amounts of granzymes suggesting a pre-activated state of these cells (39). While preactivated γδ T cells are more susceptible to cell death, we examined whether gal-3 induces apoptosis in these cells because extracellular gal-3 has been described to mediate T cell death (16, 40). To investigate apoptosis, short-term activated γδ T cells were treated with the indicated concentration of gal-3 and gal-9 as a control for 24 h. Annexin-V served as a marker for apoptotic cells and annexin-V together with PI as markers for late apoptotic cells (Figure 6B). Seventy percentage of the short-term activated γδ T cells were viable (annexin-V^−^ PI^−^) after 24 h culture in medium, whereas 6% were apoptotic (annexin-V^+^ PI^−^) and 17% late apoptotic or necrotic (annexin-V^+^ PI^+^). The addition of gal-3 as well as gal-9 at a low concentration (0.1 μg/mL) for 24 h did not influence the viability of the short-term activated γδ T cells. Interestingly, the addition of 1 μg/mL gal-9 reduced the viability of the short-term activated T cells to 43%. In these cultures, the proportion of apoptotic cells increased to 9% and of late apoptotic cells to 48%. Taken together, in contrast to gal-9, gal-3 did not induce enhanced apoptosis in short-term activated γδ T cells.

### Reduced Number of Vδ2 γδ TIL Are Suppressed in Their Proliferation by Galectin-3 Releasing Autologous PDAC Cells

In view of the gal-3 effects during the interaction of PDAC cells and T cells isolated from peripheral blood of healthy donors or PDAC patients, we speculated that the number of TIL could be reduced due to a relevant amount of soluble gal-3 present in PDAC patients.

**Figure 7:**
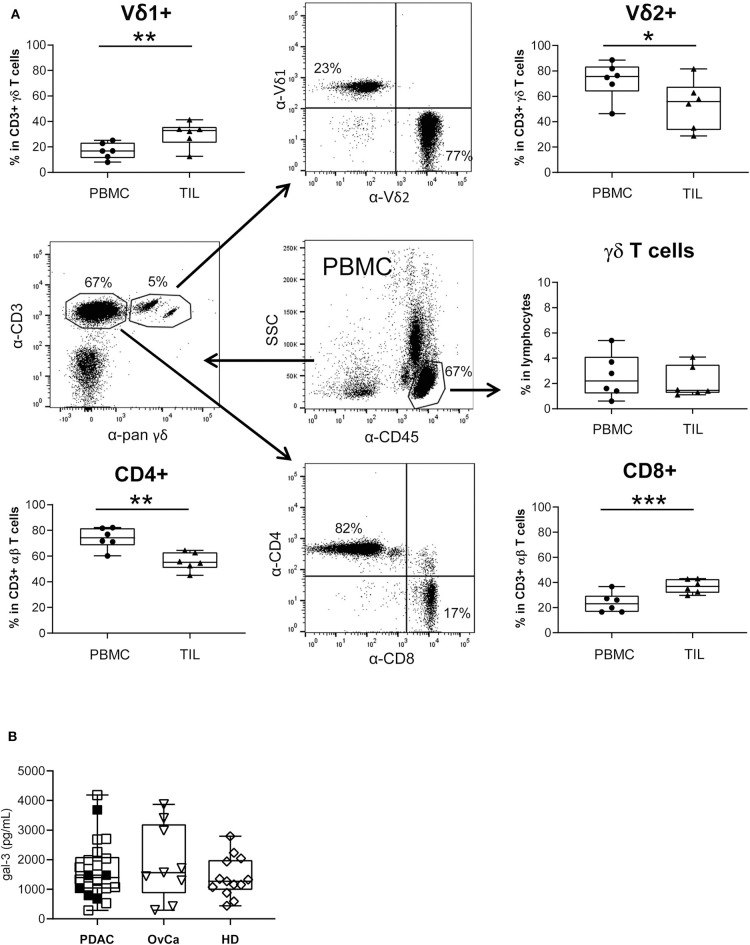
Monitoring of T lymphocyte subsets within blood and PDAC tissue, and galectin-3 serum levels. **(A)** The relative percentage of different T cell subsets within *ex vivo* isolated PBMC and TIL of PDAC patients (*n* = 6) was determined by staining the cells with the indicated mAbs and analyzed by LSR Fortessa. A gate was set on lymphocytes based on the side scatter properties and CD45 leukocytes to analyze γδ T cells within leukocytes. To distinguish between Vδ1 and Vδ2 within the CD3/pan γδ TCR, a gate was set on CD3/pan γδ TCR-expressing T cells. For discrimination between CD4 and CD8 αβ T cells a gate was set on CD3 T cells excluding CD3/pan γδ TCR^+^ cells. The gating strategy is shown with PBMC of one patient. Each symbol presents the data of one donor, and the lines in the boxes represent the median of different independent experiments. Statistical comparison of matched samples was carried out parametrically by using paired, two-tailed *t*-test. *P*-value; *P < 0.05, **P < 0.01, ***P < 0.001. As γδ T cell samples did not follow a normal distribution, Wilcoxon non-parametric, matched-pairs signed rank test was applied. *P*-value n.s., non-significant. **(B)** Gal-3 concentrations in serum samples from PDAC patients (*n* = 22), Ovarian cancer patients (OvCa, *n* = 9) and age-matched healthy donors (HD, *n* =13) measured by ELISA are presented as dot plots. The filled dots are the patients presented also in **(A)**. Samples present no significant differences.

By comparative immune profiling of TIL and PBMC of the same PDAC patients, we observed a slight decrease of the CD3 γδ T cell percentage within TIL compared to PBMC (Figure 7A). Additionally, we demonstrated an inversion of the Vδ1/Vδ2 T cell ratio within CD3 γδ T cells when we compared the percentage of the γδ T cell subsets of PBL and TIL from the same donor. Similar to the inversion of the Vδ1/Vδ2 T cell ratio, there was also an inversion of CD8/CD4 T cells within CD3 αβ T cells. We observed a significant increase of Vδ1 T cells and CD8 αβ T cells within TIL compared to PBMC, whereas Vδ2 TIL and the CD4 αβ TIL significantly decreased in comparison to PBMC from the same donor (Figure 7A). The distribution of CD8 and CD4 αβ T cells was calculated within the CD3 T cell populations by excluding pan γδ T cells.

Analyzing circulating gal-3 in serum of patients with PDAC or advanced ovarian cancer as well as in serum of age-matched healthy donors revealed that gal-3 concentrations did not differ between these groups (Figure 7B).

**Figure 8:**
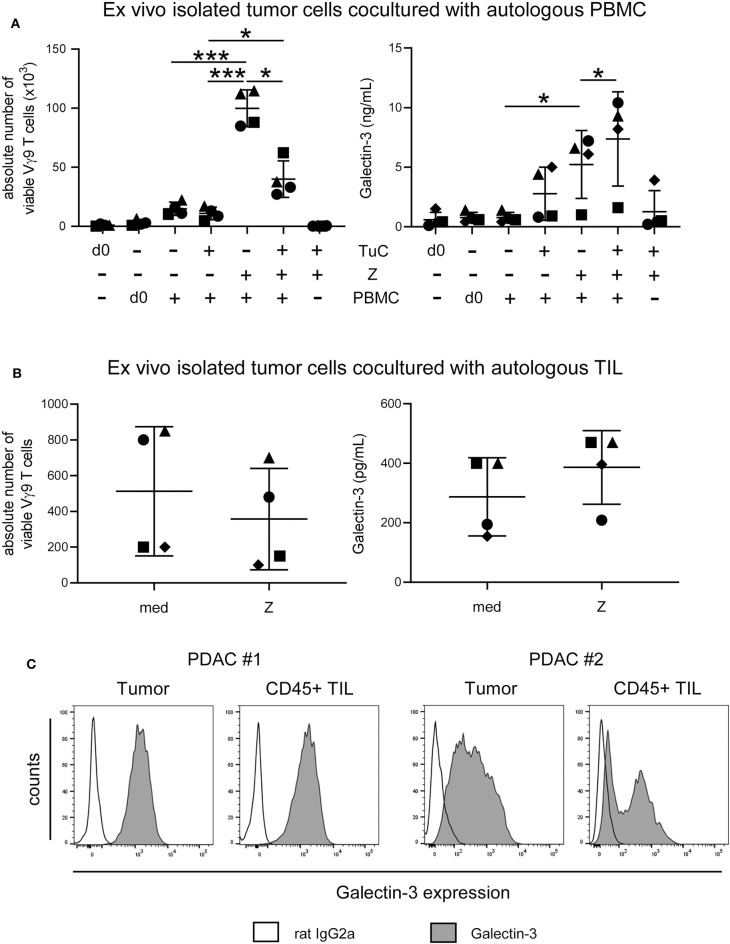
Coculture of *ex vivo* isolated PDAC cells with autologous PBMC or TIL inhibits γδ T cell proliferation and enhances release of galectin-3. **(A)** In total, 5 × 10^3^ or **(B)** 1.5 × 10^5^ *ex vivo* isolated tumor cells (TuC) and **(A)** 2.5 × 10^5^ autologous PBMC or **(B)** 1.5 × 10^3^ autologous TIL were cultured alone or together in complete medium or were stimulated with 2.5 μM zoledronic acid with 50 IU/mL rIL-2 (Z). At day 0 and 11 days after culture, the absolute cell number of the Vγ9 γδ T cells was determined using SCDA. Cell culture supernatants were collected after 96 h and galectin-3 was determined by ELISA. Each symbol presents the data of one donor, and the lines represent the median of 4 different independent experiments. **(A)** Statistical comparison of matched samples was carried out parametrically by using paired, two-tailed *t*-test. *P*-value; *P < 0.05, ***P < 0.001. **(B)** Wilcoxon non-parametric, matched-pairs signed rank test (left panel) or parametric, matched-pairs, two-tailed *t*-test (right panel) was carried out. Samples present no significant differences. **(C)** Histograms are showing intracellular gal-3 expression stained with anti-gal-3 Ab (gray) compared to the appropriate isotype-control (unfilled) in pan-Cytokeratin^+^ tumor cells and CD45^+^ leukocytes of 2 representative donors (PDAC #1 and #2) out of 4.

Since gal-3 serum levels did not differ between PDAC patients and healthy donors, we asked whether *ex vivo* isolated tumor cells release enhanced amounts of gal-3 after interaction with autologous PBMC or TIL of PDAC patients, and thereby inhibit γδ T cell proliferation. The stimulation of PBMC with zoledronic acid and IL-2 in the absence of autologous tumor cells induced a significant and selective outgrowth of Vγ9 T cells and PBMC released very low amounts of gal-3 (Figure 8A). By coculturing PBMC with autologous tumors cells without further stimulation, Vγ9 T cells did not proliferate, and gal-3 release was slightly enhanced in comparison to PBMC monoculture in the absence of tumor cells (Figure 8A). However, after stimulating the cocultured cells with zoledronic acid, γδ T cell proliferation was significantly inhibited and a significantly enhanced gal-3 release was observed (Figure 8A). We next asked if the proliferation of autologous γδ TIL is affected in a similar way as γδ PBMC of the same donor. Therefore, we cultured *ex vivo* isolated tumor cells with autologous TIL, which comprises tumor-associated cells, in IL-2 medium, and stimulated the culture with zoledronic acid or medium (Figure 8B). Interestingly, the absolute cell number of Vγ9 T cells within the TIL population was reduced after zoledronic acid stimulation and, again, we observed an increase in gal-3 release after addition of zoledronic acid to cocultured cells as well as a strong intracellular gal-3 expression in tumor cells and TIL in this autologous system (Figures 8B,C). Taken together, the absolute number of viable Vγ9 T cells within the TIL population was reduced comparable to the inhibition of Vγ9 γδ T cells within PBMC of the same donor after coculture with zoledronic acid-stimulated autologous tumor cells. Interestingly, zoledronic acid-stimulated autologous tumor cells cocultured with TIL released an enhanced amount of gal-3 compared to the medium control.

## Discussion

While an overexpression of gal-3 in PDAC tissues and a gal-3-mediated suppression of CD8 TILs have been already described, the gal-3 concentrations in the serum of pancreatic cancer patients is discussed controversially (8, 9, 41, 42). This study demonstrated that gal-3 is not increased in the serum of PDAC patients, but it is highly expressed in *ex vivo* isolated PDAC cells. In addition, gal-3 is not only expressed by PDAC cells but also by T cells within PBMC and TIL. While gal-3 is only released in small amounts by either cell population, the coculture of both populations significantly enhanced the release of gal-3. Interestingly, the stimulation of the cocultured cells further increased gal-3 release which could be of high relevance for clinical studies. Knockdown of gal-3 in PDAC cells demonstrated that PDAC cells are the main source of gal-3. Gal-3 is released by the majority of PDAC cells after interaction with T cells. It effectively inhibited T cell proliferation, but not T cell cytotoxicity regardless of presence of bsAb, which in turn potently enhanced γδ T cell cytotoxicity. We demonstrated that extracellular gal-3, released by PDAC cells after coculture with γδ T cells, binds glycosylated γδ T cell surface receptor α3β1 integrin, and contributes to inhibition of γδ T cell proliferation. This is of great interest for an *in vivo* application of PAg or zoledronic acid where the γδ T cell proliferation might be prevented if gal-3 is present.

Since the therapeutic options of PDAC treatment are very limited, and the efficacy of chemotherapeutic agents (e.g., gemcitabine) is unsatisfactory (10, 11), new therapeutic approaches including application of bsAb or silencing of gal-3 in PDAC cells could be of high clinical relevance (23, 30, 43, 44). BsAb are designed to target T cells to tumor cells, thereby enhancing T cell cytotoxicity against tumor cells. In contrast to PAg, bsAb significantly increased γδ T cell cytotoxicity against PDAC cells by inducing enhanced amounts of granzymes (23, 38). Additionally, we observed that bsAb did not further enhance gal-3 release of PDAC cells cocultured with γδ T cells, in contrast to PAg stimulation. Since the used bsAb are not designed to significantly enhance T cell proliferation, the low number of Vγ9 T cells within the tumors might be a problem. However, our preliminary results suggest that tribody [(HER2)_2_xVγ9] can potently enhance cytotoxic activity of low numbers of Vγ9 T cells with TILs, which coexpress Vδ2 as well as Vδ1 (unpublished observation). Since Vδ1 T cells are enriched within TIL of PDAC patients, the impact of gal-3 on Vδ1 T cells is of high interest. Unfortunately, the antigens which selectively induce a Vδ1 T cell proliferation are so far unknown. Therefore, Vδ1 T cell expansion within PBMC or TIL cannot be examined.

Regarding gal-3 as a potential novel target for PDAC therapy, a transient siRNA mediated silencing of gal-3 in PDAC cells suppresses their migration and invasion decreasing β-catenin, which represents an important tumor cell invasion signal (45). In addition, silencing of gal-3 in PDAC cells inhibits their proliferation and invasion, and reduced tumor size and volume in an orthotopic PDAC mouse model (13). The reports provide clear evidence for gal-3 being an important player in PDAC progression. In our study, we focused more on the role of gal-3 in the interaction of PDAC cells and T cells with special focus on γδ T cells, which infiltrate PDAC tissues, similarly to CD8 αβ T cells (22–24, 35). All T cell subsets including αβ and γδ T cells were inhibited in their proliferation after coculture with gal-3 secreting PDAC cells, independent of the T cell activation state. Recently, Kouo and colleagues reported about a gal-3-mediated inhibition in a CD8 T cell-based immunotherapy which aims to enhance the anti-tumor response of cytotoxic CD8 T lymphocytes. PDAC patients who respond to a granulocyte-macrophage colony-stimulating factor (GMC-SF)-secreting allogenic PDAC vaccine developed neutralizing gal-3 Ab after immunization. Gal-3 binds activated-committed CD8 T cells only in the tumor microenvironment and suppresses their anti-tumor response *via* Lymphocyte-activation gene (LAG)-3 and by inhibiting the expansion of plasmacytoid dendritic cells (42). Interestingly, gal-3 is described to contribute to a defect in cytokine-secretion by CD8 TIL which mediates an anergic state of these TIL due to defective actin rearrangement and a disturbed triggering of Lymphocyte function-associated antigen (LFA)-1 (αLβ2 integrin or CD11a/CD18) at the immunological synapse (46). In their previous publications, Demotte et al. observed a loss of colocalization of TCR and CD8 molecules on CD8 T cells that have low ability to bind tetramers and a reduced cytokine release (20, 47, 48). Galectin-glycoprotein lattices inhibited release but not intracellular cytokine expression by affecting actin regulators Coronin 1a and Cdc42 Rho GTPase and LFA-1 at the synapse (46).

Since gal-3 at physiological concentrations did not induce cell death in γδ T cells in our experiments, we hypothesized that, similar to CD8 T cells, gal-3 has an immunosuppressive function by inducing anti-proliferative signaling and consequently γδ T cell anergy. However, treatment with physiological gal-3 concentrations did not significantly modulate IFN-γ release or degranulation of γδ T cells, and the pre-treatment with lactose did not restore the gal-3-mediated inhibition of γδ T cell proliferation. Therefore, we suggest a different mechanism of gal-3 on γδ T cell proliferation (within PBMC or TIL). Knockdown of gal-3 in PDAC cells as well as neutralizing anti-CD49c CD29 (α3β1 integrin) mAbs partially restored gal-3-mediated inhibition of γδ T cell proliferation. Integrins such as LFA-1 binding to the intercellular adhesion molecule (ICAM, CD54) play an important role in the γδ T cell cytotoxicity toward PDAC cells (49). However, the role of CD49c/CD29 in the effector function of γδ T cells is less clear (50). CD49c/CD29 was expressed on the cell surface of resting γδ T cells, and was downregulated after activation (51). LFA-1, CD49a/CD29 (α1β1 integrin) and CD49c/CD29 are described to bind gal-3 (37, 52). The binding of gal-3 to CD49c/CD29 seems to be dependent on the glycosylation of the glycosyltransferase by β1,6-N-Acetylglucosaminyltransferase V (Mgat5) (53). Mgat5 is responsible for generating branched N-glycans that can be elongated with N-acetyl-D-lactosamine (LacNAc) sequences which bind the TCR. Gal-3 which also binds to LacNAc can compete with TCR binding thereby inhibiting TCR clustering (15). Knockdown of Mgat5 in mice prevents binding of gal-3 at the TCR, and improved the assembly of CD3 and TCR, and thereby T cell proliferation (54). Additionally, the interaction between poly LacNAc and gal-3 reduced the affinity of MHC class-I related chain (MIC) A to NK cell receptor Natural-killer group 2, member D (NKG2D), which impaired NK cell cytotoxicity against tumor cells (21). Beside CD49c/CD29, gal-3 can also bind to the γδ TCR or NKG2D expressed on Vδ2 γδ T cells, and thereby increase the threshold for γδ T cell activation. This results in an inhibition of T cell proliferation.

Chang et al. reported that α3β1 and α6β1 integrins mediate laminin/merosin binding and function as costimulatory molecules for human thymocyte proliferation (55). Our results suggest that the binding of gal-3 to α3β1 integrin prevents the proliferation-promoting effect of CD49c/CD29 on γδ T cells. A blockade of the gal-3 α3β1 integrin interaction mediated by applying a CD49c/CD29 mAb could also have an impact on the proliferation-promoting effect of α3β1 integrin. This fact together with binding of gal-3 to the TCR and/or NKG2D could be an explanation for the incomplete restoration of the γδ T cell proliferation after coculture with gal-3 secreting PancTu-I cells after application of a neutralizing CD49c/CD29 mAb.

For the release of gal-3 by PancTu-I cells a direct cell contact with T cells was necessary, which underscores the high importance to examine the influence of cell-cell interactions. In this study, the gal-3 intensity in the cell periphery of the PancTu-I cells was decreased 1–3 min after interaction with γδ T cells, and then increased again within the next 45 min. These data suggest that stored gal-3 can be released very rapidly by tumor cells after interaction with T cells and, thereafter, relocalization of gal-3 toward the cell periphery is required. Interestingly, very few synapses were formed between PDAC cells and γδ T cells during this process indicating that gal-3 hindered the formation of synapses between PDAC cells and T cells or a “kiss and run mechanism” is involved in which γδ T cells bind briefly to the tumor cell, kill it and move to the next tumor cell.

Since gal-3 has no signal sequence, the release *via* the endoplasmic reticulum/trans-golgi network is very unlikely (56). Furthermore, inhibitors of the classic secretory pathway such as brefeldin A and monensin did also not inhibit the gal-3 release by kidney cells suggesting that other mechanisms are involved (57). In this context, the release by proteolysis or by exosomes could be possible mechanisms. Gal-3 was already identified in exosomes of tumor cells (58). Matrix metalloproteinases-2 and−9, which both cleave gal-3, could release gal-3 bound to receptors or extracellular matrix (59, 60). Exosomes are an important component in immunological synapses between T cells and antigen-presenting cells (61). An association of gal-3 with ALIX at the immunological synapse was shown in Jurkat T cells. ALIX is a protein that is involved in the formation of exosomes (62, 63). Therefore, we analyzed the localization of gal-3 which was observed in vesicles of PDAC cells. To characterize these vesicles in more detail, the colocalization of gal-3 with the vesicular marker proteins CD107a (LAMP-1), CD63 (LAMP-3), Rab11 and Vti1b was examined. Gal-3 has been described in macrophages as a binding partner of the lysosomal membrane-associated protein CD107a (64). However, our results indicate that gal-3 did only slightly colocalize with CD107a in PDAC cells. Differences in the colocalization of gal-3 and CD107a in PDAC cells compared to macrophages may be due to different glycosylation patterns of CD107a in both cell populations. Different glycosylation patterns of CD107a associated with a change in gal-3 colocalization has been shown during the maturation of immature to mature dendritic cells (DC) (65). In addition, gal-3 slightly colocalizes with other vesicular marker proteins such as lysosomal membrane-associated protein CD63 and vesicle synaptosome-associated protein receptor Vti1b expressed on vesicles of the trans-golgi network or late endosomes was observed in only a fraction of cells, but not with Rab11 expressed on recycling endosomes. In other studies, gal-3 was detected in exosomes from DCs as well as from bladder carcinoma cells, which suggests that the gal-3 containing vesicles in PDAC cells may contain or resemble exosomes (58, 66).

For PDAC, survival rates have not changed significantly in the recent years and also treatment with immune checkpoint inhibitors did not achieve improvements as observed in other tumor entities (67). To enhance overall responsiveness of immunotherapy, several clinical trials have started to combine treatment with immune checkpoint inhibitors together with galectin-3 inhibitor DG-MD-02 or GR-MD02 to enhance therapeutic effects in other tumor entities (42). Thus, another promising approach is the usage of bsAb which are able to restore T cell cytotoxicity against PDAC cells of previously non-reactive T cells. Our results indicate that gal-3 has an immunosuppressive function on the proliferation of circulating as well as tumor-infiltrating T cells and that T cell cytotoxicity against PDAC cells can be significantly enhanced by bsAb.

BsAb targeting γδ T cells provide a tool to enhance cytotoxic capacity of γδ T cells, and gal-3 inhibitors to overcome suppression of proliferation. Clinical studies are certainly required to further investigate the therapeutic potential of combing bsAb and galectin inhibitors.

## Data Availability Statement

The raw data supporting the conclusions of this article will be made available by the authors, without undue reservation.

## Ethics Statement

In accordance with the Declaration of Helsinki, written informed consent was obtained from all donors, and the research was approved by the relevant institutional review boards (Ethic Committee of the Medical Faculty of the CAU Kiel, code number: D405/10, D445/18, and A110/99).

## Author Contributions

DG, H-HO, ML, and DW performed experiments. DG, H-HO, and DW designed the study with the help of DK. SS provided blood, serum, and tissue from PDAC patients. DB provided serum from advanced ovarian cancer patients. MP designed and provided the bispecific antibodies. DG and H-HO analyzed the data and designed the figures. DK, SS, DB, and MP contributed to the discussion. DW designed the project and wrote and finalized the manuscript. All authors critically reviewed the manuscript.

## Conflict of Interest

The authors declare that the research was conducted in the absence of any commercial or financial relationships that could be construed as a potential conflict of interest.

## Abbreviations

bsAb: bispecific antibody
BrHPP: bromohydrinpyrophosphate
CRD: conserved carbohydrate recognition domains
DC: dendritic cell
ERK: Extracellular-signal Regulated Kinases
gal-3: galectin-3
gal-9: galectin-9
HER-2: Human epidermal growth factor receptor-2
LacNAc: N-acetyl-D-lactosamine
mAb: monoclonal antibody
MICA: MHC class-I related chain A
n-BP: aminobisphosphonate
NKG2D: natural-killer group 2, member D
PDAC: pancreatic ductal adenocarcinoma
PI: propidium iodide
Ras: rat sarcoma
TCR: T cell receptor
TIL: tumor-infiltrating lymphocytes
PAg: phosphorylated antigen
PBMC: peripheral blood mononuclear cells
UKSH: University Hospital Schleswig-Holstein.

## References

[1] Dumic J Dabelic S Flogel M. Galectin-3: an open-ended story. Biochim Biophys Acta. (2006) 1760:616–35. 10.1016/j.bbagen.2005.12.020 16478649

[2] Radosavljevic G Volarevic V Jovanovic I Milovanovic M Pejnovic N Arsenijevic N. The roles of Galectin-3 in autoimmunity and tumor progression. Immunol Res. (2012) 52:100–10. 10.1007/s12026-012-8286-6 22418727

[3] Newlaczyl AU Yu LG. Galectin-3–a jack-of-all-trades in cancer. Cancer Lett. (2011) 313:123–8. 10.1016/j.canlet.2011.09.003 21974805

[4] Song L Tang JW Owusu L Sun MZ Wu J Zhang J. Galectin-3 in cancer. Clin Chim Acta. (2014) 431:185–91. 10.1016/j.cca.2014.01.019 24530298

[5] Rabinovich GA Baum LG Tinari N Paganelli R Natoli C Liu FT Galectins and their ligands: amplifiers, silencers or tuners of the inflammatory response? Trends Immunol. (2002) 23:313–20. 10.1016/S1471-4906(02)02232-9 12072371

[6] Hann A Gruner A Chen Y Gress TM Buchholz M. Comprehensive analysis of cellular galectin-3 reveals no consistent oncogenic function in pancreatic cancer cells. PLoS ONE. (2011) 6:e20859. 10.1371/journal.pone.0020859 21698183 PMC3116838

[7] Luo Z Wang Q Lau WB Lau B Xu L Zhao L. Tumor microenvironment: the culprit for ovarian cancer metastasis? Cancer Lett. (2016) 377:174–82. 10.1016/j.canlet.2016.04.038 27131957

[8] Schaffert C Pour PM Chaney WG. Localization of galectin-3 in normal and diseased pancreatic tissue. Int J Pancreatol. (1998) 23:1–9. 9520085 10.1007/BF02787497

[9] Xie L Ni WK Chen XD Xiao MB Chen BY He S. The expressions and clinical significances of tissue and serum galectin-3 in pancreatic carcinoma. J Cancer Res Clin Oncol. (2012) 138:1035–43. 10.1007/s00432-012-1178-2 22367363 PMC11824273

[10] Hidalgo M Cascinu S Kleeff J Labianca R Lohr JM Neoptolemos J. Addressing the challenges of pancreatic cancer: future directions for improving outcomes. Pancreatology. (2015) 15:8–18. 10.1016/j.pan.2014.10.001 25547205

[11] Siegel RL Miller KD Jemal A Cancer statistics 2019. CA Cancer J Clin. (2019) 69:7–34. 10.3322/caac.21551 30620402

[12] Ji B Tsou L Wang H Gaiser S Chang DZ Daniluk J. Ras activity levels control the development of pancreatic diseases. Gastroenterology. (2009) 137:1072–82. 10.1053/j.gastro.2009.05.052 19501586 PMC2789008

[13] Song S Ji B Ramachandran V Wang H Hafley M Logsdon C. Overexpressed galectin-3 in pancreatic cancer induces cell proliferation and invasion by binding Ras and activating Ras signaling. PLoS ONE. (2012) 7:e42699. 10.1371/journal.pone.0042699 22900040 PMC3416861

[14] Elad-Sfadia G Haklai R Balan E Kloog Y. Galectin-3 augments K-Ras activation and triggers a Ras signal that attenuates ERK but not phosphoinositide 3-kinase activity. J Biol Chem. (2004) 279:34922–30. 10.1074/jbc.M312697200 15205467

[15] Cagnoni AJ Perez Saez JM Rabinovich GA Marino KV. Turning-off signaling by siglecs, selectins, and galectins: chemical inhibition of glycan-dependent interactions in Cancer. Front Oncol. (2016) 6:e109. 10.3389/fonc.2016.00109 27242953 PMC4865499

[16] Stillman BN Hsu DK Pang M Brewer CF Johnson P Liu FT. Galectin-3 and galectin-1 bind distinct cell surface glycoprotein receptors to induce T cell death. J Immunol. (2006) 176:778–89. 10.4049/jimmunol.176.2.778 16393961

[17] Fukumori T Takenaka Y Yoshii T Kim HR Hogan V Inohara H. CD29 and CD7 mediate galectin-3-induced type II T-cell apoptosis. Cancer Res. (2003) 63:8302–11. 14678989

[18] Peng W Wang HY Miyahara Y Peng G Wang RF. Tumor-associated galectin-3 modulates the function of tumor-reactive T cells. Cancer Res. (2008) 68:7228–36. 10.1158/0008-5472.CAN-08-1245 18757439 PMC3181121

[19] Xue H Liu L Zhao Z Zhang Z Guan Y Cheng H. The N-terminal tail coordinates with carbohydrate recognition domain to mediate galectin-3 induced apoptosis in T cells. Oncotarget. (2017) 8:49824–38. 10.18632/oncotarget.17760 28548942 PMC5564810

[20] Demotte N Stroobant V Courtoy PJ Van Der Smissen P Colau D Luescher IF. Restoring the association of the T cell receptor with CD8 reverses anergy in human tumor-infiltrating lymphocytes. Immunity. (2008) 28:414–24. 10.1016/j.immuni.2008.01.011 18342010

[21] Tsuboi S Sutoh M Hatakeyama S Hiraoka N Habuchi T Horikawa Y. A novel strategy for evasion of NK cell immunity by tumours expressing core2 O-glycans. EMBO J. (2011) 30:3173–85. 10.1038/emboj.2011.215 21712812 PMC3160189

[22] Helm O Mennrich R Petrick D Goebel L Freitag-Wolf S Roder C. Comparative characterization of stroma cells and ductal epithelium in chronic pancreatitis and pancreatic ductal adenocarcinoma. PLoS ONE. (2014) 9:e94357. 10.1371/journal.pone.0094357 24797069 PMC4010424

[23] Oberg HH Peipp M Kellner C Sebens S Krause S Petrick D. Novel bispecific antibodies increase gammadelta T-cell cytotoxicity against pancreatic cancer cells. Cancer Res. (2014) 74:1349–60. 10.1158/0008-5472.CAN-13-0675 24448235

[24] Oberg HH Grage-Griebenow E Adam-Klages S Jerg E Peipp M Kellner C. Monitoring and functional characterization of the lymphocytic compartment in pancreatic ductal adenocarcinoma patients. Pancreatology. (2016) 16:1069–79. 10.1016/j.pan.2016.07.008 27424476

[25] Wrobel P Shojaei H Schittek B Gieseler F Wollenberg B Kalthoff H. Lysis of a broad range of epithelial tumour cells by human gamma delta T cells: involvement of NKG2D ligands and T-cell receptor- versus NKG2D-dependent recognition. Scand J Immunol. (2007) 66:320–8. 10.1111/j.1365-3083.2007.01963.x 17635809

[26] Espinosa E Belmant C Pont F Luciani B Poupot R Romagne F. Chemical synthesis and biological activity of bromohydrin pyrophosphate, a potent stimulator of human gamma delta T cells. J Biol Chem. (2001) 276:18337–44. 10.1074/jbc.M100495200 11279081

[27] Gober HJ Kistowska M Angman L Jeno P Mori L De LG Human T cell receptor gammadelta cells recognize endogenous mevalonate metabolites in tumor cells. J Exp Med. (2003) 197:163–8. 10.1084/jem.20021500 12538656 PMC2193814

[28] Oberg HH Kellner C Peipp M Sebens S Adam-Klages S Gramatzki M. Monitoring circulating gammadelta T cells in cancer patients to optimize gammadelta T cell-based immunotherapy. Front Immunol. (2014) 5:e643. 10.3389/fimmu.2014.00643 25566256 PMC4269191

[29] Oberg HH Kellner C Gonnermann D Peipp M Peters C Sebens S. gammadelta T cell activation by bispecific antibodies. Cell Immunol. (2015) 296:41–9. 10.1016/j.cellimm.2015.04.009 25979810

[30] Oberg HH Kellner C Gonnermann D Sebens S Bauerschlag D Gramatzki M. Tribody [(HER2)2xCD16] is more effective than trastuzumab in enhancing γ*δ* T cell and natural killer cell cytotoxicity against HER2-expressing Cancer Cells. Front Immunol. (2018) 9:e814. 10.3389/fimmu.2018.00814 29725336 PMC5916959

[31] Gonnermann D Oberg HH Kellner C Peipp M Sebens S Kabelitz D. Resistance of cyclooxygenase-2 expressing pancreatic ductal adenocarcinoma cells against γ*δ* T cell cytotoxicity. Oncoim. (2014) 4:e988640. 10.4161/2162402X.2014.988460 25949900 PMC4404835

[32] Sipos B Moser S Kalthoff H Torok V Lohr M Kloppel G. A comprehensive characterization of pancreatic ductal carcinoma cell lines: towards the establishment of an *in vitro* research platform. Virchows Arch. (2003) 442:444–52. 10.1007/s00428-003-0784-4 12692724

[33] Pechhold K Pohl T Kabelitz D. Rapid quantification of lymphocyte subsets in heterogeneous cell populations by flow cytometry. Cytometry. (1994) 16:152–9. 10.1002/cyto.990160209 7924684

[34] Janssen O Wesselborg S Heckl-Ostreicher B Pechhold K Bender A Schondelmaier S. T cell receptor/CD3-signaling induces death by apoptosis in human T cell receptor gamma delta+T cells. J Immunol. (1991) 146:35–9. 1824593

[35] Daley D Zambirinis CP Seifert L Akkad N Mohan N Werba G. γ*δ* T cells support pancreatic oncogenesis by restraining alphabeta T cell activation. Cell. (2016) 166:1485–99. 10.1016/j.cell.2016.07.046 27569912 PMC5017923

[36] Tawfik D Groth C Gundlach JP Peipp M Kabelitz D Becker T. TRAIL-Receptor 4 modulates gammadelta T cell-cytotoxicity toward cancer cells. Front Immunol. (2019) 10:e2044. 10.3389/fimmu.2019.02044 31555275 PMC6722211

[37] Fukushi J Makagiansar IT Stallcup WB. NG2 proteoglycan promotes endothelial cell motility and angiogenesis via engagement of galectin-3 and alpha3beta1 integrin. Mol Biol Cell. (2004) 15:3580–90. 10.1091/mbc.e04-03-0236 15181153 PMC491820

[38] Jonescheit H Oberg HH Gonnermann D Hermes M Sulaj V Peters C. Influence of indoleamine-2,3-dioxygenase and its metabolite kynurenine on gammadelta T cell cytotoxicity against ductal pancreatic adenocarcinoma cells. Cells. (2020) 9:E1140. 10.3390/cells9051140 32384638 PMC7290398

[39] Oberg HH Janitschke L Sulaj V Weimer J Gonnermann D Hedemann N. Bispecific antibodies enhance tumor-infiltrating T cell cytotoxicity against autologous HER-2-expressing high-grade ovarian tumors. J Leukoc Biol. (2019) 9:1071–8. 10.1002/JLB.5MA1119-265R 31833593 PMC7318294

[40] Yang RY Hsu DK Liu FT. Expression of galectin-3 modulates T-cell growth and apoptosis. Proc Natl Acad Sci USA. (1996) 93:6737–42. 10.1073/pnas.93.13.6737 8692888 PMC39096

[41] Gaida MM Bach ST Gunther F Baseras B Tschaharganeh DF Welsch T. Expression of galectin-3 in pancreatic ductal adenocarcinoma. Pathol Oncol Res. (2012) 18:299–307. 10.1007/s12253-011-9444-1 21910036

[42] Kouo T Huang L Pucsek AB Cao M Solt S Armstrong T. Galectin-3 shapes antitumor immune responses by suppressing CD8+ T cells via LAG-3 and inhibiting expansion of plasmacytoid dendritic cells. Cancer Immunol Res. (2015) 3:412–23. 10.1158/2326-6066.CIR-14-0150 25691328 PMC4390508

[43] Chou FC Chen HY Kuo CC Sytwu HK. Role of galectins in tumors and in clinical immunotherapy. Int J Mol Sci. (2018) 19:e430. 10.3390/ijms19020430 29389859 PMC5855652

[44] Sun Q Zhang Y Liu M Ye Z Yu X Xu X. Prognostic and diagnostic significance of galectins in pancreatic cancer: a systematic review and meta-analysis. Cancer Cell Int. (2019) 19:e309. 10.1186/s12935-019-1025-5 31832021 PMC6873495

[45] Kobayashi T Shimura T Yajima T Kubo N Araki K Tsutsumi S. Transient gene silencing of galectin-3 suppresses pancreatic cancer cell migration and invasion through degradation of beta-catenin. Int J Cancer. (2011) 129:2775–86. 10.1002/ijc.25946 21448903 PMC3833077

[46] Petit AE Demotte N Scheid B Wildmann C Bigirimana R Gordon-Alonso M. A major secretory defect of tumour-infiltrating T lymphocytes due to galectin impairing LFA-1-mediated synapse completion. Nat Commun. (2016) 7:e12242. 10.1038/ncomms12242 27447355 PMC4961845

[47] Demotte N Wieers G Van Der Smissen P Moser M Schmidt C Thielemans K. A galectin-3 ligand corrects the impaired function of human CD4 and CD8 tumor-infiltrating lymphocytes and favors tumor rejection in mice. Cancer Res. (2010) 70:7476–88. 10.1158/0008-5472.CAN-10-0761 20719885

[48] Demotte N Bigirimana R Wieers G Stroobant V Squifflet JL Carrasco J. A short treatment with galactomannan GM-CT-01 corrects the functions of freshly isolated human tumor-infiltrating lymphocytes. Clin Cancer Res. (2014) 20:1823–33. 10.1158/1078-0432.CCR-13-2459 24526733

[49] Liu Z Guo B Lopez RD Expression of intercellular adhesion molecule (ICAM)-1 or ICAM-2 is critical in determining sensitivity of pancreatic cancer cells to cytolysis by human gammadelta-T cells: implications in the design of gammadelta-T-cell-based immunotherapies for pancreatic cancer. J Gastroenterol Hepatol. (2009) 24:900–11. 10.1111/j.1440-1746.2008.05668.x 19175829

[50] Siegers GM. Integral roles for integrins in gammadelta T cell function. Front Immunol. (2018) 9:e521. 10.3389/fimmu.2018.00521 29593745 PMC5859029

[51] Avdalovic M Fong D Formby B. Adhesion and costimulation of proliferative responses of human gamma delta T cells by interaction of VLA-4 and VLA-5 with fibronectin. Immunol Lett. (1993) 35:101–8. 10.1016/0165-2478(93)90077-F 8509148

[52] Ochieng J Leite-Browning ML Warfield P. Regulation of cellular adhesion to extracellular matrix proteins by galectin-3. Biochem Biophys Res Commun. (1998) 246:788–91. 10.1006/bbrc.1998.8708 9618290

[53] Saravanan C Liu FT Gipson IK Panjwani N. Galectin-3 promotes lamellipodia formation in epithelial cells by interacting with complex N-glycans on alpha3beta1 integrin. J Cell Sci. (2009) 122(Pt 20):3684–93. 10.1242/jcs.045674 19755493 PMC2758802

[54] Demetriou M Granovsky M Quaggin S Dennis JW. Negative regulation of T-cell activation and autoimmunity by Mgat5 N-glycosylation. Nature. (2001) 409:733–9. 10.1038/35055582 11217864

[55] Chang AC Salomon DR Wadsworth S Hong MJ Mojcik CF Otto S. Alpha 3 beta 1 and alpha 6 beta 1 integrins mediate laminin/merosin binding and function as costimulatory molecules for human thymocyte proliferation. J Immunol. (1995) 154:500–10. 7814863

[56] Sato S Burdett I Hughes RC. Secretion of the baby hamster kidney 30-kDa galactose-binding lectin from polarized and nonpolarized cells: a pathway independent of the endoplasmic reticulum-Golgi complex. Exp Cell Res. (1993) 207:8–18. 10.1006/excr.1993.1157 8319774

[57] Lindstedt R Apodaca G Barondes SH Mostov KE Leffler H. Apical secretion of a cytosolic protein by Madin-Darby canine kidney cells. Evidence for polarized release of an endogenous lectin by a nonclassical secretory pathway. J Biol Chem. (1993) 268:11750–7. 8505302

[58] Welton JL Khanna S Giles PJ Brennan P Brewis IA Staffurth J. Proteomics analysis of bladder cancer exosomes. Mol Cell Proteomics. (2010) 9:1324–38. 10.1074/mcp.M000063-MCP201 20224111 PMC2877990

[59] Ochieng J Fridman R Nangia-Makker P Kleiner DE Liotta LA Stetler-Stevenson WG. Galectin-3 is a novel substrate for human matrix metalloproteinases-2 and−9. Biochemistry. (1994) 33:14109–14. 10.1021/bi00251a020 7947821

[60] Ochieng J Green B Evans S James O Warfield P. Modulation of the biological functions of galectin-3 by matrix metalloproteinases. Biochim Biophys Acta. (1998) 1379:97–106. 10.1016/S0304-4165(97)00086-X 9468337

[61] Choudhuri K Llodra J Roth EW Tsai J Gordo S Wucherpfennig KW. Polarized release of T-cell-receptor-enriched microvesicles at the immunological synapse. Nature. (2014) 507:118–23. 10.1038/nature12951 24487619 PMC3949170

[62] Baietti MF Zhang Z Mortier E Melchior A Degeest G Geeraerts A. Syndecan-syntenin-ALIX regulates the biogenesis of exosomes. Nat Cell Biol. (2012) 14:677–85. 10.1038/ncb2502 22660413

[63] Chen HY Fermin A Vardhana S Weng IC Lo KF Chang EY. Galectin-3 negatively regulates TCR-mediated CD4+ T-cell activation at the immunological synapse. Proc Natl Acad Sci USA. (2009) 106:14496–501. 10.1073/pnas.0903497106 19706535 PMC2732795

[64] Dong S Hughes RC. Macrophage surface glycoproteins binding to galectin-3 (Mac-2-antigen). Glycoconj J. (1997) 14:267–74. 9111144 10.1023/a:1018554124545

[65] Bax M Garcia-Vallejo JJ Jang-Lee J North SJ Gilmartin TJ Hernandez G. Dendritic cell maturation results in pronounced changes in glycan expression affecting recognition by siglecs and galectins. J Immunol. (2007) 179:8216–24. 10.4049/jimmunol.179.12.8216 18056365

[66] Thery C Boussac M Veron P Ricciardi-Castagnoli P Raposo G Garin J. Proteomic analysis of dendritic cell-derived exosomes: a secreted subcellular compartment distinct from apoptotic vesicles. J Immunol. (2001) 166:7309–18. 10.4049/jimmunol.166.12.7309 11390481

[67] Bisht S Feldmann G. Novel targets in pancreatic cancer therapy - current status and ongoing translational efforts. Oncol Res Treat. (2018) 41:596–602. 10.1159/000493437 30269126

