# Supplementary figures and images for: Galectin-3 Released by Pancreatic Ductal Adenocarcinoma Suppresses γδ T Cell Proliferation but Not Their Cytotoxicity
Source: Front Immunol. 2020 Jun 30;11:1328. doi: 10.3389/fimmu.2020.01328 (PMC7338555; doi:10.3389/fimmu.2020.01328)

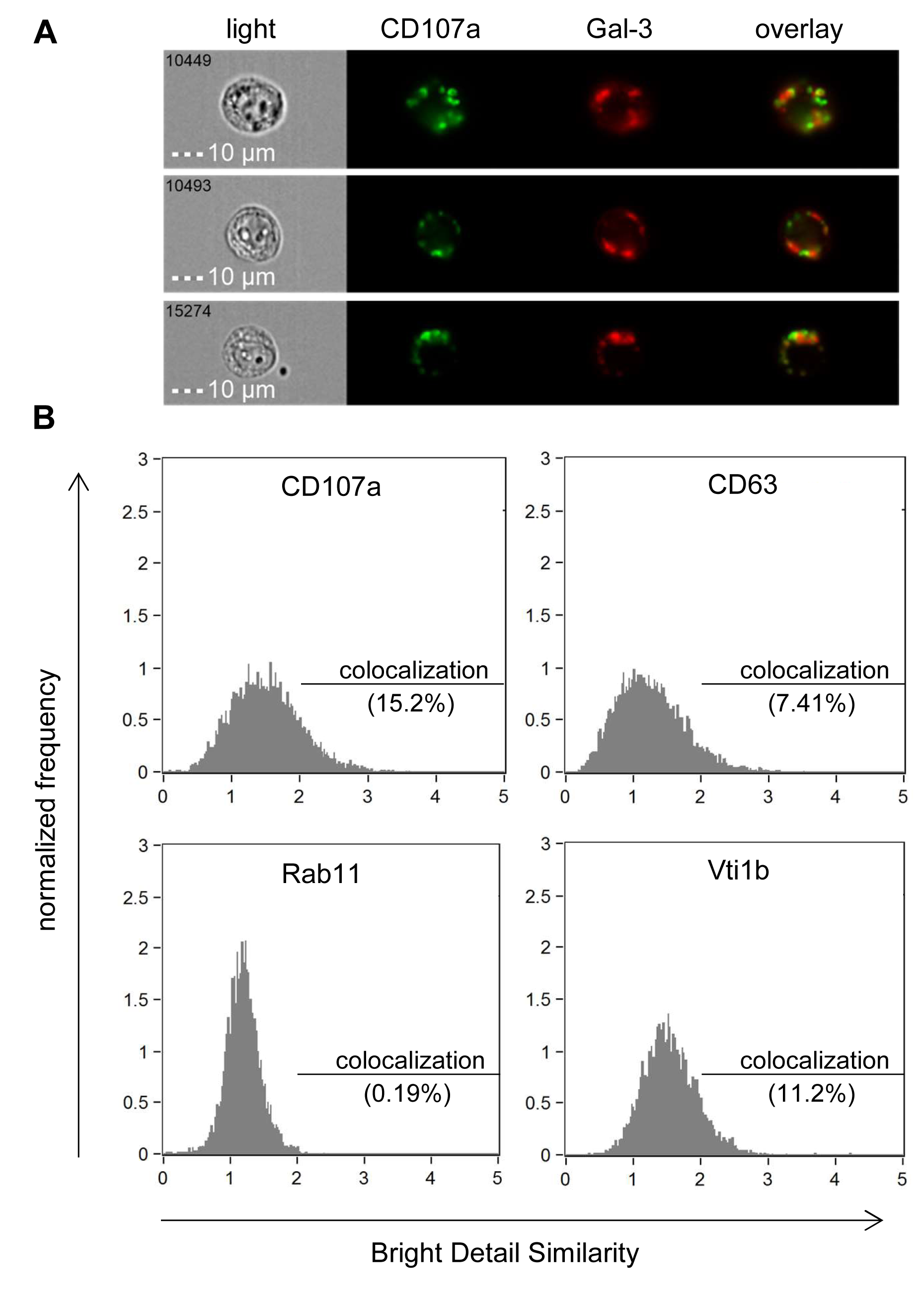

Supplement: Supplementary file 1 [file Image_1.TIF]

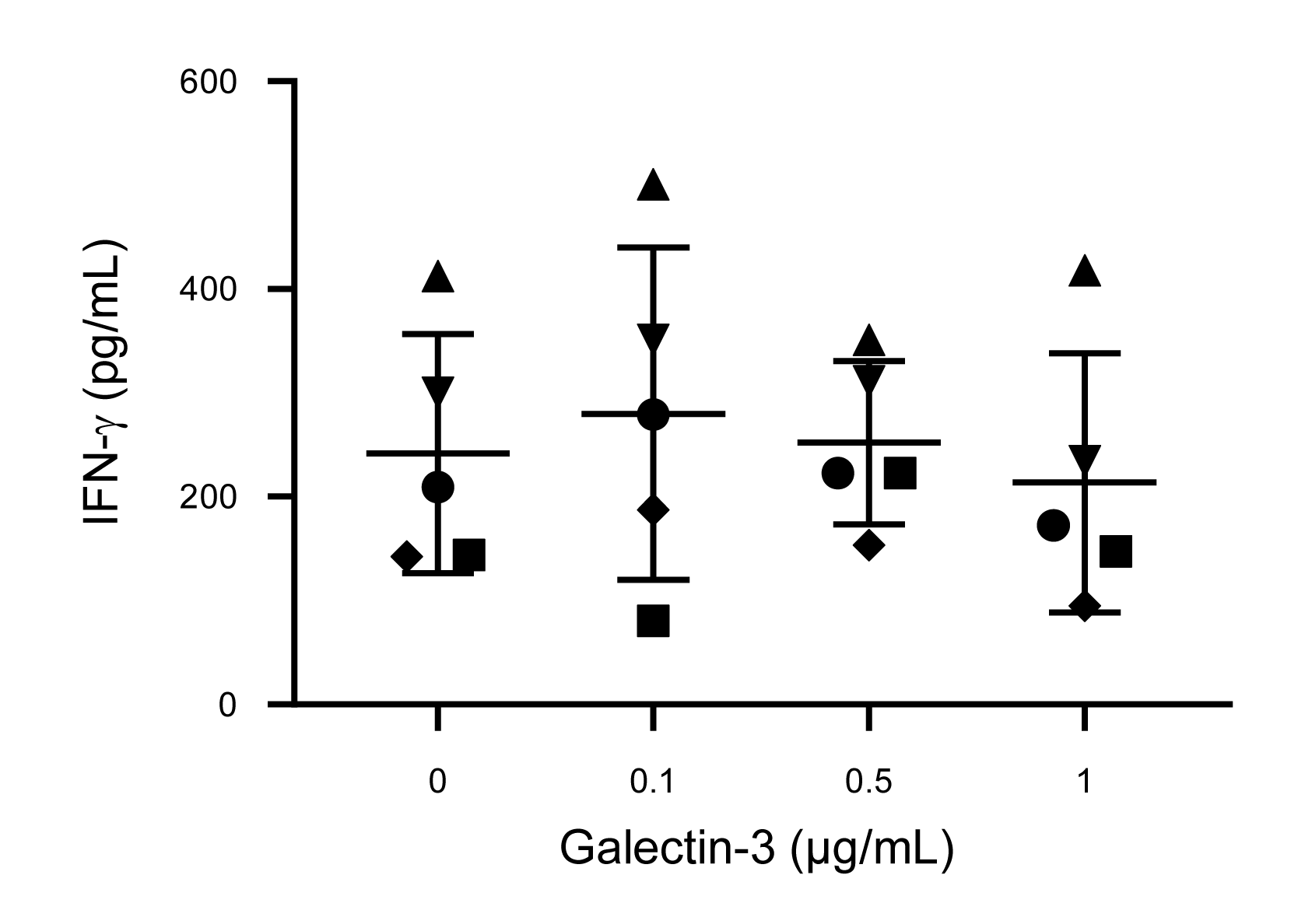

Supplement: Supplementary file 2 [file Image_2.TIF]

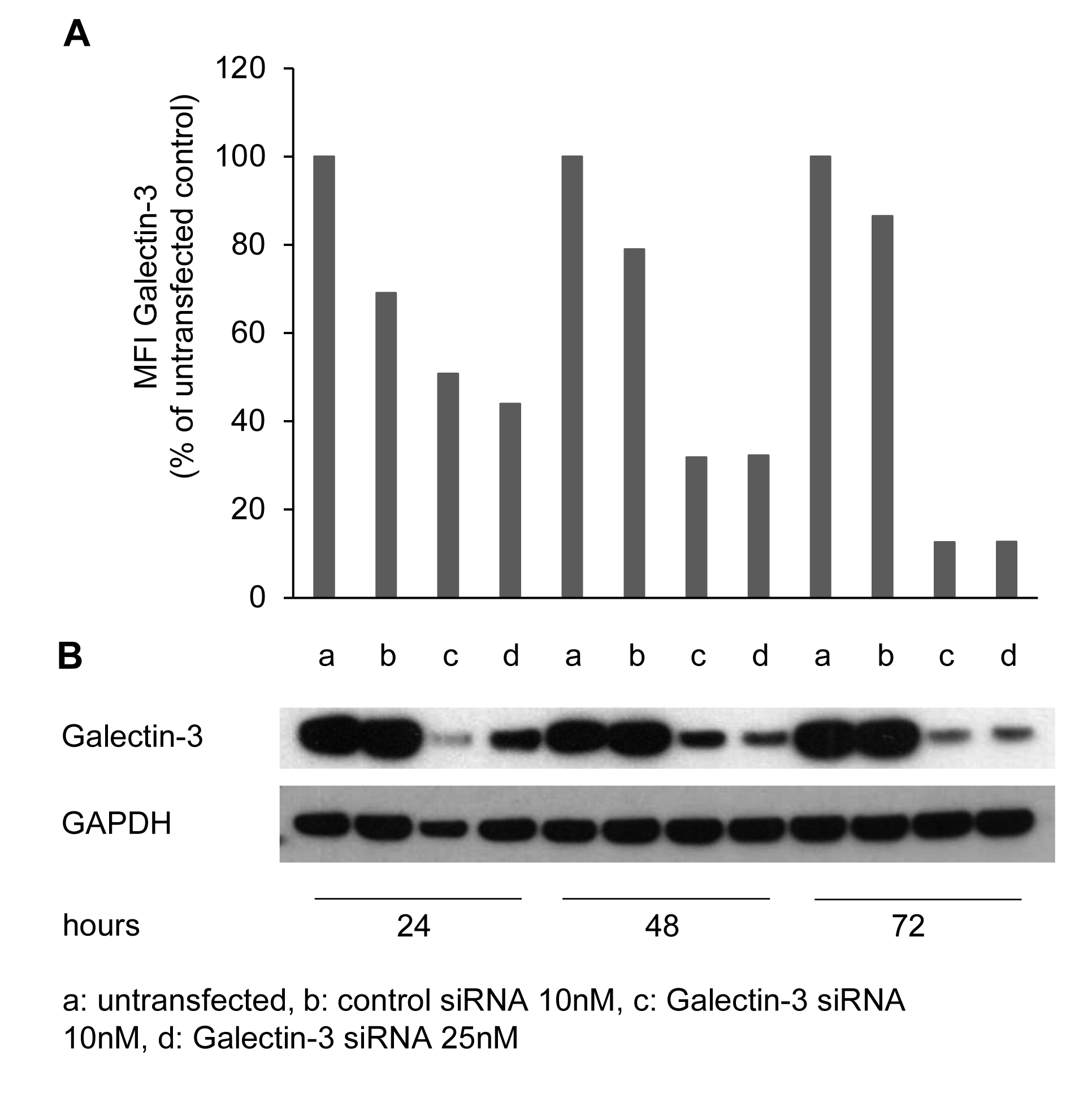

Supplement: Supplementary file 3 [file Image_3.TIF]

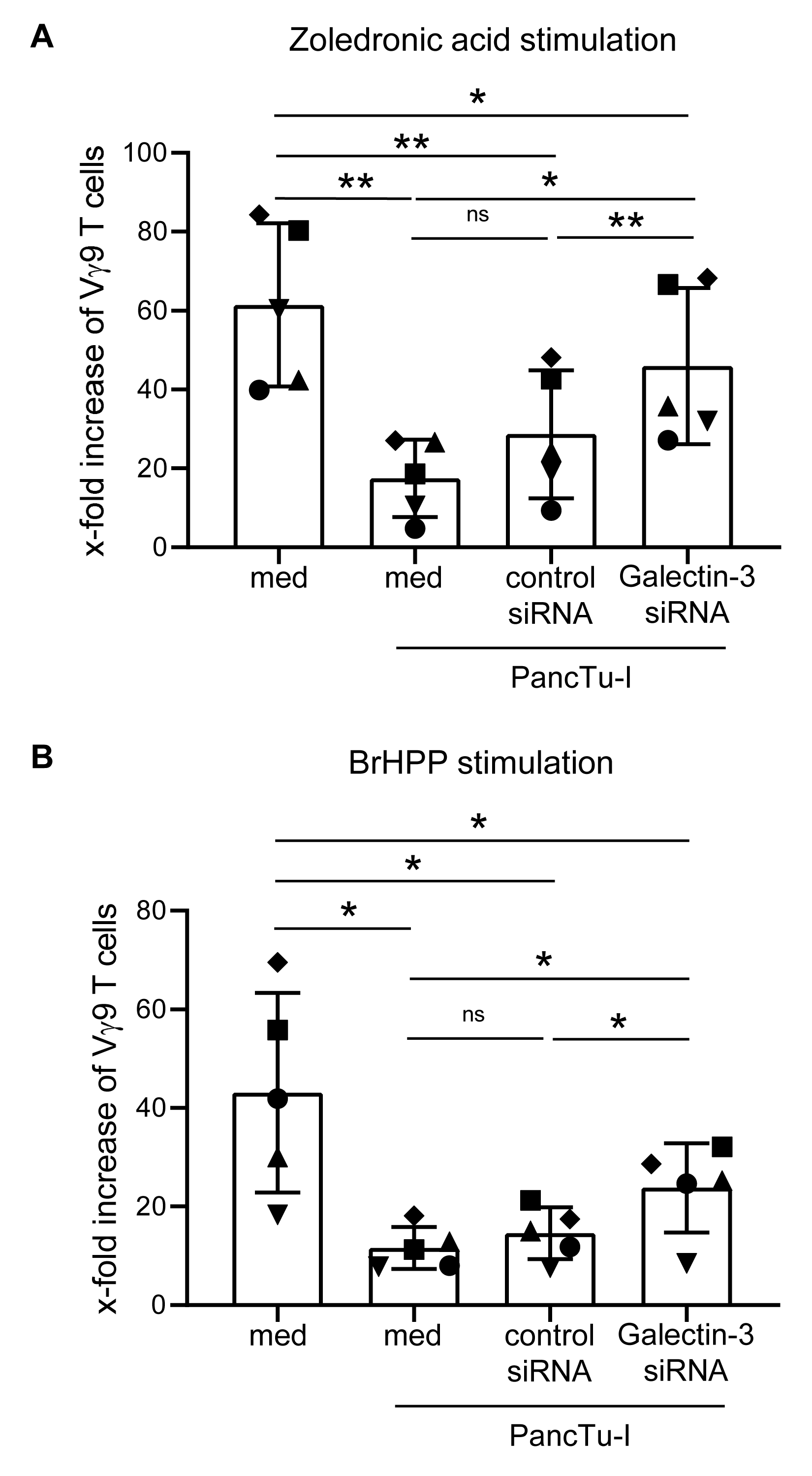

Supplement: Supplementary file 4 [file Image_4.TIF]

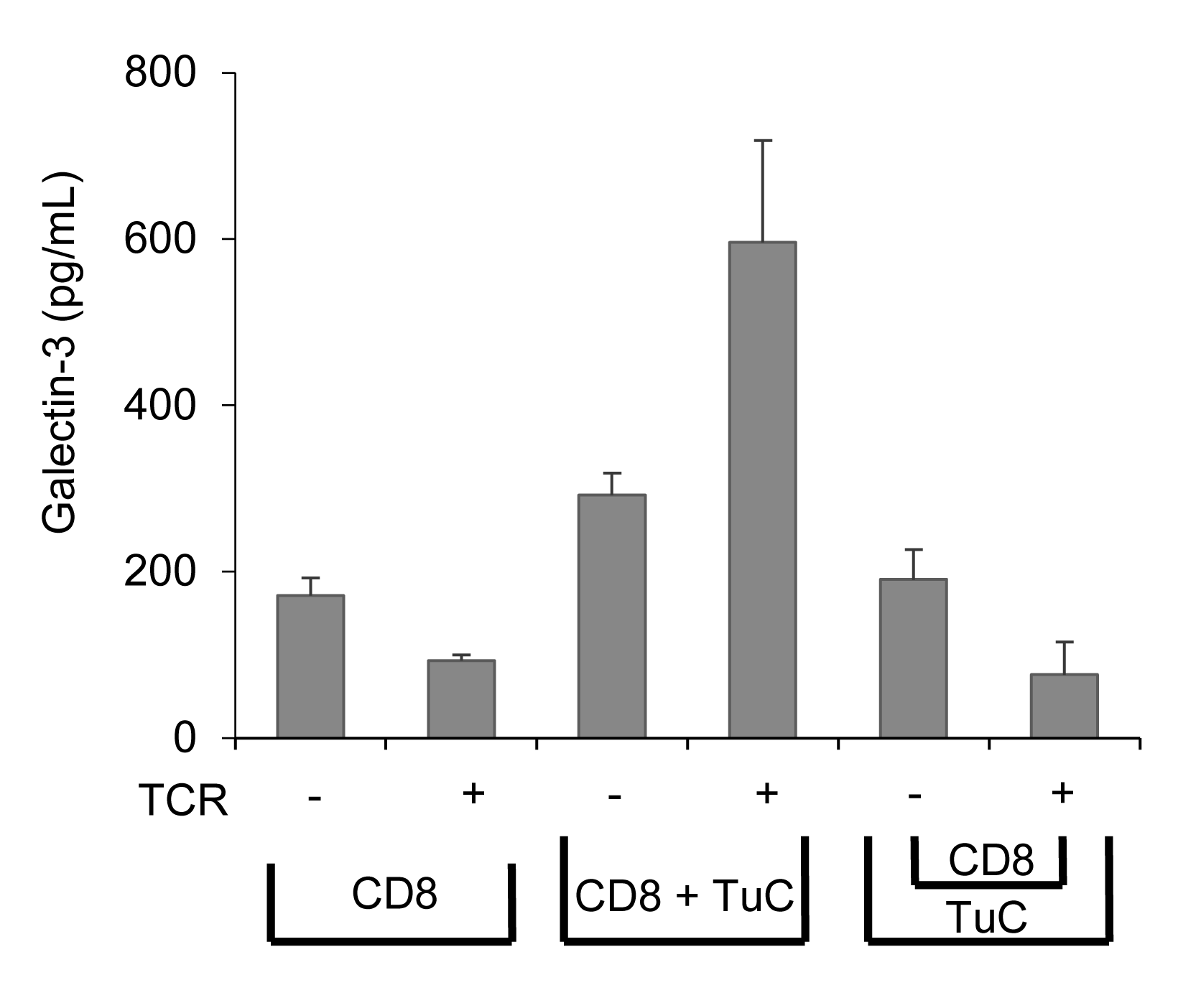

Supplement: Supplementary file 5 [file Image_5.TIF]

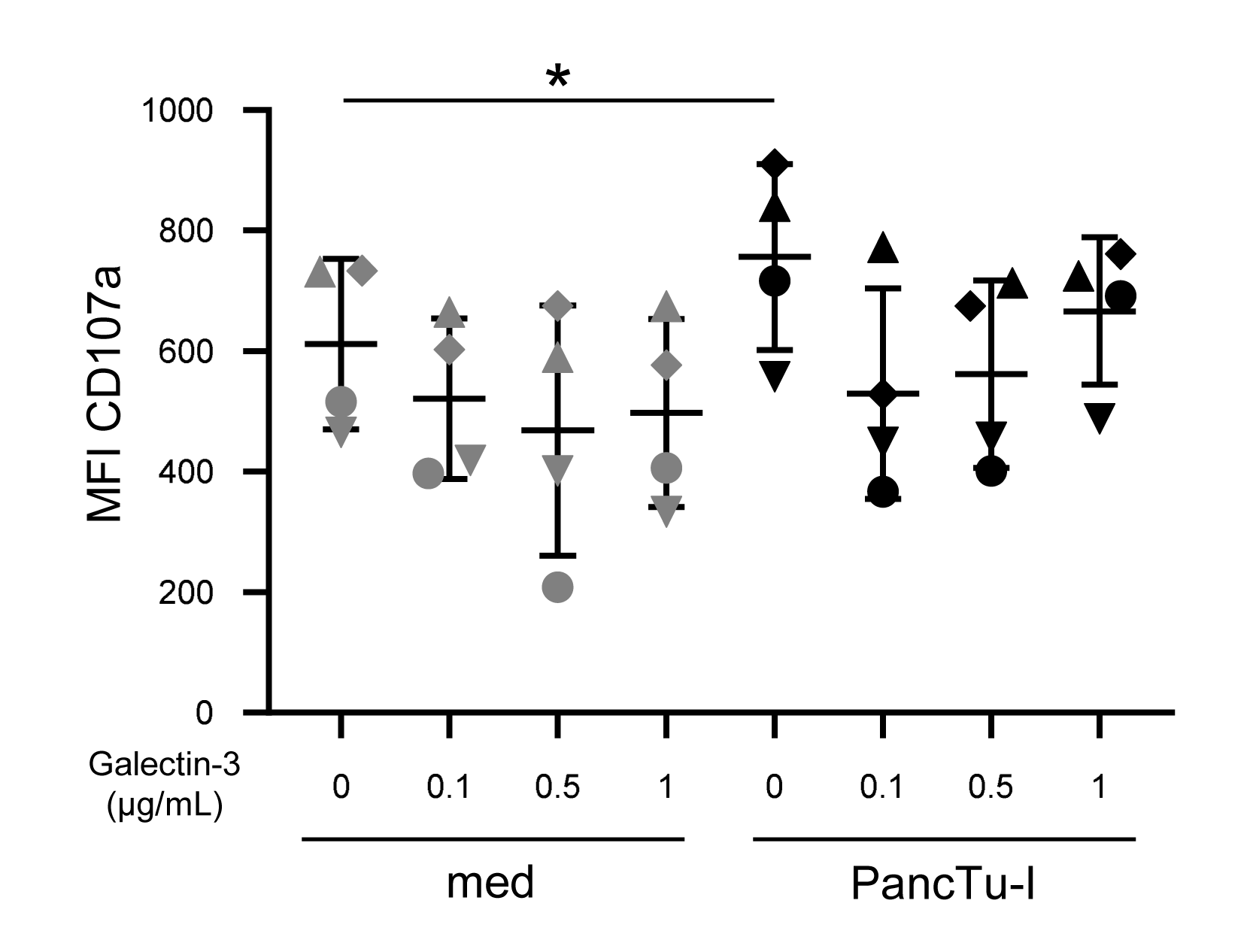

Supplement: Supplementary file 6 [file Image_6.TIF]
