# Supplementary material for: Galectin-3 Released by Pancreatic Ductal Adenocarcinoma Suppresses γδ T Cell Proliferation but Not Their Cytotoxicity
Source: Front Immunol. 2020 Jun 30;11:1328. doi: 10.3389/fimmu.2020.01328 (PMC7338555; doi:10.3389/fimmu.2020.01328)
Supplement: Supplementary file 7 [file Data_Sheet_1.pdf]

Supplemental data

Supplemental Materials and Methods:

Western blot analysis

PDAC cells were lysed in Nonidet P-40 lysis buffer (Fluka Chemie, Buchs, Switzerland) with 1% (v/v) detergent in 50 mM Tris-HCl, 150 mM NaCl, 2 mM EDTA with protease and phosphatase inhibitors sodium fluoride, sodium orthovanadate and the complete protease inhibitor mix™ (Roche, Mannheim, Germany). Samples were separated on 4-12% Bis-Tris NuPAGE Gels (Thermo Fisher Scientific), and protein was transferred to nitrocellulose membranes (Hybond C-Extra, GE Healthcare, Munich, Germany). Blots were blocked with 5% BSA, and gal-3 was detected by 1-10 µg/mL purified mouse anti-gal-3 Ab clone Gal397 (BioLegend) followed by HRP-conjugated sheep-anti-mouse secondary Ab (GE Healthcare). Membranes were stripped and reprobed with anti-GAPDH Ab clone 6C5 (1:3000, Santa Cruz, Dallas, USA) as a control for loading and transfer. Proteins were visualized by the enhanced chemiluminescence system (GE Healthcare).

Imaging flow cytometry

To analyze colocalization of gal-3 with the vesicular marker proteins CD107a (LAMP-1), CD63 (LAMP-3) (both are lysosomal membrane-associated proteins), Vti1b (vesicle synaptosome-associated protein receptor, v-SNARE) expressed on vesicles of the trans-golgi network or late endosomes and Rab11 (expressed on recycling endosomes), cells were intracellularly stained with Ab as follows: 1 µg/mL anti-gal-3 clone M3/38 (BioLegend) followed by 10 µg/mL AF647-conjugated chicken anti-rat secondary antibody (Thermo Fisher Scientific) together with 1.2 µg/mL PE-conjugated anti-CD107a clone H4A3 (BioLegend) or 25 µg/mL PE-labeled anti-CD63 clone MEM-259 (Abcam, Cambridge, UK) or combined with 5 µg/mL anti-Vti1b clone 7 (BD Biosciences) followed by 10 µg/mL

AF555-conjugated donkey anti-mouse secondary Ab (Thermo Fisher Scientific) or 1 µg/mL anti-Rab11 followed by 10 µg/mL AF555-conjugated donkey anti-rabbit secondary Ab (both from Thermo Fisher Scientific). These images were then analyzed with the Bright Detail Similarity algorithm using the IDEAS® image analysis software to quantify the degree of colocalization in double-positive cells. This algorithm represents the log-transformed Pearson correlation of respective images on a pixel-by-pixel and cell-by-cell basis, whereby Bright Detail Similarity scores > 2 indicate colocalization.

IFN-γ ELISA

In total, 10^5 PBMC in 100 µL of complete medium with 50 IU/ mL rIL-2 were cultured in 96-well microtiter plates (Thermo Fisher Scientific). γδ T cells within PBMC were selectively activated by 300 nM phosphorylated antigen (PAg) bromohydrinpyrophosphate (BrHPP, Innate Pharma, Marseille, France) in the absence or presence of different concentrations (0.1, 0.5 and 1 µg/mL) of gal-3 (BioLegend, San Diego, CA, USA) for 48 h. Supernatants were collected and stored at -20°C until use. Human IFN-γ was determined in duplicates using ELISA by human IFN-γ DuoSet ELISA kit (R&D Systems, Wiesbaden, Germany) following the procedures outlined by the manufacturer.

CD107a degranulation assay

In total, 5 x 10^5 PancTu-I cells in 50 µL complete medium were cultured in 96-well flat-bottom plates (Nunc) overnight. After 24 h, 50 µL short-term activated Vγ9Vδ2 γδ T cells in complete medium with 12.5 IU/mL rIL-2 and 300 nM BrHPP were added at an E/T ratio of 25:1 to the indicated PDAC cells. Alternatively, Vγ9Vδ2 γδ T cells alone were stimulated with 300 nM BrHPP and 12.5 IU/mL rIL-2. 50 µL of different concentrations (0.1, 0.5 and 1 µg/mL) of gal-3 (BioLegend) or medium were added as indicated. For CD107a assay, 10 µL PE-labeled anti-human CD107a mAb clone H4A3 (50 µg/mL, BioLegend) or appropriate

isotype control was added directly to the culture, whereas 3 µM monensin (Merck) was added 1 h after coculturing the cells. After additional 3 h, Vγ9Vδ2 γδ T cells were stained with AF488-labeled anti-Vγ9 mAb [clone 7A5, (34)], and analyzed by flow cytometry.

Supplemental Legends to Figures:

Suppl. Fig. 1. Galectin-3 weakly colocalizes with CD107a, CD63 or Vti1b but not with Rab11. The vesicular marker proteins CD107a, CD63, Rab11 and Vti1b as well as gal-3 were intracellularly stained in PancTu-I cells and the colocalization was analyzed by the ImageStream® X Mark II. (A) Bright field images of 3 representative PDAC cells are shown on the left, and appropriate fluorescence images of the staining and the overlay of CD107a and gal-3 are shown on the right. (B) A possible colocalization of gal-3 with CD107a, CD63, Rab11 and Vti1b was analyzed with the Bright Detail Similarity feature of the IDEAS® software in 6-7 x10^3 PDAC cells. The number in brackets indicates the proportion of cells with a Bright Detail Similarity of more than 2.

Suppl. Fig. 2. Galectin-3 does not significantly modulate IFN-γ release of γδ T cells within PBMC. In total, 1 x 10^5 PBMC of donors (n = 5) were stimulated with 300 nM BrHPP with the different indicated concentrations of rgal-3 and 50 IU/mL rIL-2. IFN-γ release was determined in the supernatant after 48 h using ELISA. Each symbol represents the data of one donor, and the lines represent median values of 4 different independent experiments. Statistical comparison of matched samples was carried out parametrically by using paired, two-tailed t-test. Samples present no significant differences.

Suppl. Fig. 3. Galectin-3 can be downregulated by using siRNA. PancTu-I cells were cultivated being (a) non-transfected or (b) transfected with 10 nM control siRNA, (c) 10 nM and (d) 25 nM gal-3 siRNA. After 24, 48 and 72 h, the tumor cells were trypsinized and the

gal-3 content was analyzed by (A) flow cytometry or by (B) Western blot. (A) The mean fluorescence intensity (MFI) of gal-3 is shown as a percentage of the non-transfected cells. (B) After separation of the proteins in a gel and transfer to a nitrocellulose membrane, this was first developed with an antibody against gal-3, then with an antibody against GAPDH and corresponding secondary antibodies.

Suppl. Fig. 4. Galectin-3 knockdown in PancTu-I cells partially restores γδ T cell proliferation. (A, B) In total, 5 x 10^3 PancTu-I cells left non-transfected or transfected with either control siRNA or gal-3 siRNA were cultured in complete medium 72 h after their transfection. After 24 h, 2.5 x 10^5 PBMC were added (E/T 50:1) or cultured alone. After addition of 50 IU/mL rIL-2, cells were left unstimulated (med) or stimulated with (A) 2.5 µM zoledronic acid or (B) 300 nM BrHPP. (A, B) After 6 days, the absolute cell number of the Vγ9 γδ T cells was determined using SCDA. The x-fold increase of the absolute cell number of Vγ9 T cells cultured over 6 days compared to day 0 is shown as mean ± SD of duplicates from 5 donors. Statistical comparison of matched samples was carried out parametrically by using paired, two-tailed t-test. *P* value; * = P < 0.05, ** = P < 0.01, ns = not significant.

Suppl. Fig. 5. Galectin-3 release of PDAC cell cocultured with CD8 αβ T cells is cell contact-dependent. Short-term activated CD8 αβ T cells were cultured without tumor cells and directly cocultured with 2 x 10^4 PancTu-I cells (TuC) or indirectly separated by a semipermeable membrane with 0.4 µm pores of a transwell insert in 24-well plates at an E/T ratio of 40:1. Cells were left untreated or stimulated with Activation/Expander Beads. After 24 h, gal-3 was measured in the cell culture supernatant using ELISA. Means ± SD of duplicates from 2 donors are shown. Low number of donors does not allow statistical comparison.

Suppl. Fig. 6. Galectin-3 does not significantly modulate degranulation of γδ T cells. In total, 5 x 10^5 PancTu-I cells were cultured in complete medium overnight. Thereafter, short-term activated Vγ9Vδ2 γδ T cells in complete medium with 12.5 IU/mL rIL-2 were added at an E/T ratio of 25:1 or T cells were cultured alone (med). All cultures were stimulated with 300 nM BrHPP in the absence or presence of the indicated gal-3 concentrations. In addition, anti-human CD107a mAb or an appropriate isotype control was added to the cultures. Monensin was added 1 h after starting the culture. After 4 h, Vγ9Vδ2 γδ T cells were stained with anti-Vγ9 mAb, and analyzed by LSR-Fortessa. Statistical comparison of matched samples was carried out parametrically by using paired, two-tailed t-test. Samples present no significant differences.
